# A novel SO_2_ probe inhibits lysophagy induced by Senecavirus A infection by promoting LAMP1 Cys375 sulfenylation

**DOI:** 10.1371/journal.ppat.1013932

**Published:** 2026-02-05

**Authors:** Shuo Wang, WenWen Han, BaoXiang Zhao, Ye Hong, Jun Li, JunYing Miao, ZhaoMin Lin

**Affiliations:** 1 Shandong Provincial Key Laboratory of Development and Regeneration, School of Life Science, Shandong University, Qingdao, P.R. China; 2 Key Laboratory of Livestock and Poultry Multi-omics of MARA, Institute of Animal Science and Veterinary Medicine, Shandong Academy of Agricultural Sciences, Jinan, P.R. China; 3 Institute of Organic Chemistry, School of Chemistry and Chemical Engineering, Shandong University, Jinan, P.R. China; 4 State Key Laboratory of Microbial Technology (Microbial Technology Institute), Shandong University, Qingdao, P.R. China; 5 Institute of Medical Science, the Second Hospital of Shandong University, Jinan, P.R. China; Institute of Life Sciences, INDIA

## Abstract

Lysophagy plays a key role in maintaining autophagy homeostasis, but the induction and regulation mechanisms of lysophagy are not clear. In this study, we found that Senecavirus A (SVA) dramatically decreased lysosomal-associated membrane protein 1(LAMP1), significantly increased lysosomal permeability, and induced lysophagy. We demonstrated that the SO_2_ probe (2-(4-(dimethylamino-) phenyl)1,1, 3-trimethyl-1h-benzo [e] indole-3-ium, DLC) could inhibited the degradation of LAMP1 and reduced lysophagy caused by SVA infection. DLC directly binds to LAMP1, and enhanced sulfenylation modification of LAMP1 at Cys375 to inhibit non-lysine ubiquitination. Finally, we verified the antiviral effects of DLC in cells and in BALB/c mice. Taken together, our study lays the foundation for the identification of SVA infection targets and the development of antiviral drugs in the future.

## Author summary

Research has shown that SVA infection can induce autophagy to facilitate its own replication, but the specific type of autophagy activated remains unclear. We found that SVA infection led to the degradation of the lysosomal membrane protein LAMP1. Following lysosomal damage, lysophagy was activated to clear the impaired lysosomes. Here, we identified a novel antiviral small molecule, DLC, and through a series of experiments, revealed that DLC inhibits lysophagy and suppresses SVA replication by enhancing sulfinylation modification mediated by the gas signaling molecule SO_2_, thereby regulating the sulfinylation-ubiquitination balance. This study aims to fill the gap in understanding how SVA manipulates the lysophagy pathway and may provide potential targets for developing novel lysosome-targeting anti-SVA drugs, holding significant scientific implications.

## Introduction

Autophagy is a catabolic process in cells that can degrade and recycle damaged proteins and organelles to maintain the stability of the intracellular environment and cell survival [1]. Some viruses have evolved the ability to inhibit autophagy in order to evade degradation and immune responses. Others induce autophagy, but then hijack autophagosomes as replication sites, or hijack secretory autophagy pathways to promote maturation and outflow of viral particles, thereby improving replication and transmission efficiency [2,3].Therefore, studying the relationship between virus replication and autophagy regulation is very important for us to reveal the pathogenic mechanism of viruses and explore the effects of viruses on the physiological activities of host c

Senecavirus A (SVA) is a single-strand, positive-sense RNA virus in the family Picornaviridae. In the breeding industry, SVA infection can lead to swine vesicular lesions, which is a new infectious agent that affects the pig industry seriously. At the same time, it is also an oncolytic virus that has entered clinical trials [4,5]. At present, autophagy has different effects on SVA replication in different host cells, in different time periods after virus infection, and in different pathways. Therefore, it is necessary to identify the pathway and mode of autophagy induced by SVA infection. Studies have shown that SVA can adapt to virus invasion by regulating lysosomal permeability. The internalization of SVA virus into PK-15 cells is dependent on low pH, motility, CavME, and macropinocytosis, and ultimately transport to lysosomes via RAB5-dependent early endosomes and Rab7. Ingestion by pathogens or bacteria can completely destroy the lysosome membrane to escape into the cytosol or regulate the penetration of the host membrane with secreted components [6–8].In this case, the damaged lysosome is recognized by galectin-3(Gal3), the endo-lysosome damage response (ELDR) complex is bound by E3 ubiquitin ligase to promote the recruitment of LC3 to initiate lysophagy [9,10]. Virus-induced autophagy degradation was once thought to be a phagocytosis, which effectively reduced the intracellular load of the virus, but also triggered other types of selective autophagy with various effects on the virus. However, there are few studies on lysophagy caused by viral replication. Since the entry of SVA depends on the low pH environment of the cytoplasm, our study took the lysosome damage caused by the improvement of the permeability of the lysosome membrane after SVA virus invaded host cells as the entry point to explore the detailed activity and mechanism of autophagy induced by the virus into host cells.

Lysosomes are acidic membrane-bound organelles rich in hydrolases that are responsible for the degradation of macromolecules from the extracellular space through endocytosis or phagocytosis, and from the cytoplasm through autophagy [11]. Lysosome associated membrane proteins (LAMPs) are integrated membrane glycoproteins that are primarily present in lysosomes and are involved in a variety of cellular functions [12]. LAMPs may be involved in the interaction and fusion of lysosomes with themselves and with other cellular components, including endosomes, phagosomes, and plasma membranes. Overexpression of LAMP1, LAMP2, or LAMP3 allowed 293T cells to efficiently process MuV-F [13, 14]. In addition, these LAMPs were found to interact with both MuV-F and Flynn’s protease [15]. LAMP1 has been shown to play an important role in the entry of Lassa virus (LASV) and is often used as a marker protein indicating lysosomal structure and function [15]. Studies in LAMP1-deficient mice and cells have shown that LAMP1 protein is important for the fusion of lysosomes with phagosomes and autophagosomes [16]. In addition, because LAMP1 is a highly glycosylated protein, most of the studies on it have focused on glycosylation modification, while the post-translational modification of other amino acids has been poorly studied. Therefore, we investigated the occurrence of lysophagy and the effect of LAMP1 sulfenylation after SVA infection, which are crucial for revealing the mechanism of viral replication and searching for antiviral drug targets.

Cysteine sulfenylation refers to the oxidation of cysteine residues to form sulfenic acid (Cys-SOH), a reversible modification that can act as a signaling mechanism in response to oxidative stress. Sulfenylation can modulate the activity of enzymes and transcription factors, thereby influencing various biological processes, including cell signaling and stress responses [17,18].Currently, the most common detection methods use sulfonated protein-rich derivative probes, including transcription factor-based Yap1 probes, diketone based DYn-2 chemical probes, and benzothiazol-based BTD probes [19,20]. Due to the unique nature of their thiol group, cysteine residues are particularly susceptible to various post-translational modifications. Cysteine ubiquitination is a special form of post-translational modification, which plays a crucial role in protein degradation, signaling and cellular response to stress [21]. It has been shown that cysteines can be involved in D4R ubiquitination, suggesting that nonlysine ubiquitination may have a functional impact on receptor signaling and degradation [22]. At present, there are few studies on the specific mechanisms of sulfenylation and ubiquitination and their function in antivirus. Therefore, the development of related research is conducive to the in-depth understanding of various biological processes and potential therapeutic targets of diseases related to dysregulated protein modification.

In our previous work, we synthesized and characterized a novel probe DLC (The chemical structure diagram is shown in (S1 Fig) for binding intracellular HSO_3_^-^/SO_3_^2-^ [23].We also demonstrated that DLC could promote the translocation of lipid droplets(LDs)to lysosomes, promote the sulfylation level of LAMP1, promote the decomposition of lipid droplets, and inhibit high glucose-induced senescence of endothelial cells. Meanwhile, DLC has also been shown to increase the H^+^ concentration of lysosomes and protect the function of lysosomes. Therefore, the regulatory mechanism of DLC on lysosomes in different physiological states deserves our further exploration [24].

In this study, we identified the SO_2_ probe DLC, which effectively inhibits SVA-induced LAMP1 degradation through sulfenylation modification, preserves lysosomal membrane stability, and attenuates lysophagy. These effects collectively suppress SVA proliferation in host cells.

## Results

### SVA infection triggers LMP, leading to the lysophagy

**Fig 1:**
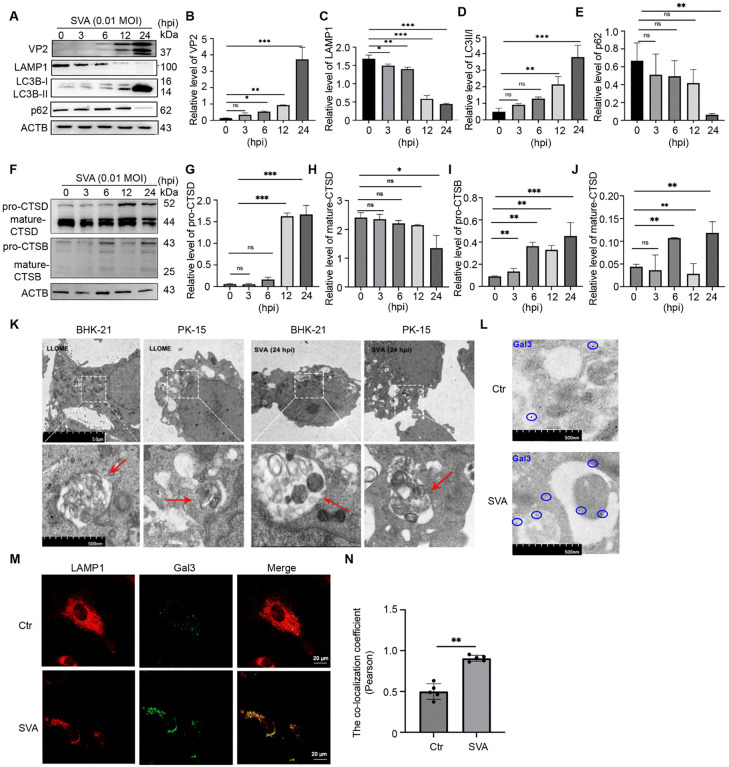
SVA infection leads to lysophagy. **(A-E)** After SVA infection, BHK-21 cells were collected at different time points, and the expression levels of lysophagy related proteins and SVA VP2 proteins were examined. **(F-J)** The cytoplasm fraction of BHK-21 cells were extracted at different time points after SVA infection, and the protein levels of CTSD and CTSB (mature and precursor) in the two fractions were detected by Western Blot.(K) Comparison between lysophagy and LLOME (1 mM) addition in BHK-21 cells and PK-15 cells infected by SVA under Transmission electron microscope. In the enlarged images, the infected and LLOME cells present a damaged lysosome with a single-layer membrane structure typical of autophagosomes compared to the control group (red arrow). Scale bar:5 μm,500 nm.(L) Cell sections from SVA infected and control groups were incubated with primary anti-Gal3 antibody.After incubation with secondary antibodies conjugated to 5nm colloidal gold particles, representative TEM images were observed and photographed to record, and the gold particles were mainly localized in the interior of the autophagic vesicles. Scale bar: 500 nm **(M,N)** Colocalization of LAMP1 with Gal3 in cells with or without SVA infection(6 hpi), five single-cell fields of view were randomly selected from each group, and the co-localization coefficients (Pearson coefficients) of the two channels were statistically analyzed. Scale bar: 20 μm. (*p < 0.05, **p < 0.01, ***p < 0.001, n = 3).

Our previous study showed that SVA infection not only elevated the autophagic flow, but also had an effect on the morphology and number of lysosomes in cells [25]. Therefore, we speculate that SVA may cause damage to the structure of lysosomes after infecting cells. By detecting the expression of autophagy marker proteins and lysosomal marker proteins in BHK-21 cells after different infection times (Fig 1A). We found that the expression of SVA VP2 protein increased with the prolongation of infection time (Fig 1B). However, the protein level of LAMP1 was decreased (Fig 1C). The expression of LC3BII/I increased with the infection time while SQSTM1/ p62 protein showed a downward trend (Fig 1D and 1E). Lysosomal membrane permeabilization (LMP) normally results in the transfer of lysosomal cathepsins from the lysosomal lumen to the cytoplasm and leads to lysosphagy [26]. In addition, we also examined the protein level changes of other lysosomal membrane proteins (LAMP2 and LAMP3) after SVA infection. The results showed that, consistent with LAMP1, LAMP2 decreased with SVA, but the overall LAMP3 protein showed an upward trend. It is suggested that the effects of SVA infection on lysosomal membrane proteins with different functions are different (S2 Fig).In the cytoplasmic fraction, the expression level of CTSD precursor protein increased in a time-dependent manner, and the expression level of mature protein decreased significantly at 24 hours. The CTSB precursor protein fluctuated slightly, but increased significantly at 24 hours after infection, while the mature protein increased at 6 hours and 24 hours after infection, and the overall trend also showed an increase. (Fig 1F-J). To demonstrate that SVA infection can cause lysosomal damage, morphological changes of lysosomes were observed under electron microscopy. We used L-leucine-L-leucine methyl ester (LLOME), an inducer of lysosomal damage, as a positive control [27]. Typical lysophagy was observed in both BHK-21 and PK-15 cells after SVA infection compared to LLOME (1 mM) treatment. The number of autophagosomes was increased and many damaged lysosomal structures were coated. Due to different degrees of autophagy, some lysosomes had been degraded into disordered membrane structures (Fig 1K).Moreover, gold particles (black dots, 5 nm) of Gal3 were mainly localized in the inner membrane of the autophagosome after SVA infection compared with the uninfected group. (Fig 1L) Next, we tested whether this injury led to the accumulation of lysophagy markers, so we examined the colocalization of LAMP1 with Galectin-3(Gal3) by confocal microscopy. The results revealed that at 6 hours after SVA infection, LAMP1 showed punctate dispersion and increased colocalization with Gal3 compared to that in control cells, further validating that lysosomal damage caused by SVA infection promoted the accumulation of lysosomal autophagy markers (Fig 1M and 1N). Similarly, this was confirmed by an increase in β-hexosaminidase (β-HEX) activity in the cell supernatant component, but a decrease in the lysosomal component (S3 Fig).

### DLC reduced lysophagy caused by SVA infection and inhibited the degradation of LAMP1

**Fig 2:**
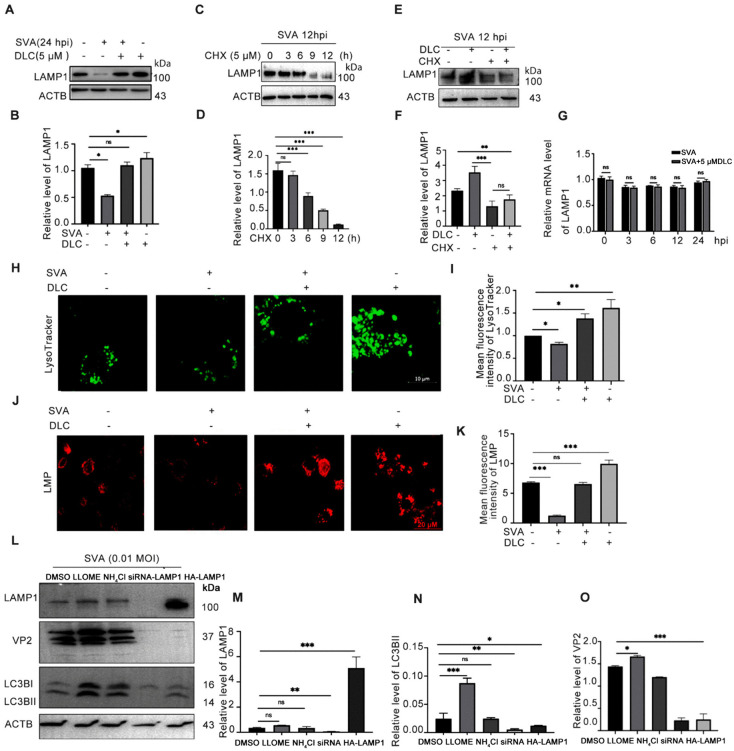
Protective effect of DLC on lysosomes after SVA infection. **(A,B)** Effect of DLC and SVA infection on the expression of LAMP1 protein. **(C,D)** LAMP1 protein levels were detected after treatment with the protein synthesis inhibitor CHX (5 μM) for 0, 3, 6, and 12 h within 12 hours of SVA infection. **(E,F)** After viral infection, CHX (5 μM) was used separately or in combination to determine the pathway by which DLC maintains LAMP1 protein. **(G)** The mRNA expression of LAMP1 in cells treated with DLC (5 μM) at different time points after infection. **(H,I)** Cells were incubated with Lysosensor Green DND-189(0.1 μM)for 1h, and the lysosome fluorescence intensity was detected under a laser confocal microscope. Three fields were selected and the average fluorescence intensity was statistically analyzed. **(J,K)** The lysosomal membrane permeability(LMP) was detected by acridine orange staining, and the red fluorescence intensity (Excitation wavelength 555nm, emission wavelength 617nm) in any three fields was counted and statistically analyzed. **(L-O)** BHK-21 cells were pretreated with DMSO, LLOME (1mM), NH_4_Cl (2 μM), over-expressed HA-LAMP1 and LAMP1 siRNA, and then infected with SVA at an MOI of 0.01 for 24h. The cell lysate was collected. The protein expression of LAMP1 and VP2 was detected. Scale bar: 10 μm, 20 μm. (*p < 0.05, **p < 0.01, ***p < 0.001, n = 3).

In our previous study, we identified a novel sulfur dioxide probe, DLC, which enhances the sulfenylation level of LAMP1. This, in turn, promotes the catabolism of lipid droplets to inhibit high glucose-induced endothelial cell senescence [24]. In the preliminary experiment, we tested by CCK-8 assay that different concentrations of DLC did not affect the activity of BHK-21 and PK-15 cells (S4 Fig).To investigate whether DLC can inhibit lysosomal damage induced by SVA virus infection, we treated infected BHK-21 cells with 5 μM DLC and observed the changes in LAMP1 as well as autophagic flux after infection under different treatment conditions. We found that DLC could inhibit the decreased expression of LAMP1 protein caused by SVA infection (Fig 2A and 2B). To determine the mechanism of the reduction of LAMP1 protein by SVA, we examined the protein level of LAMP1 in cells treated with CHX (5 μM) for different periods of time within 12 h of SVA infection and found that LAMP1 protein was further reduced with the extension of CHX treatment (Fig 2C and 2D). Furthermore, we demonstrated that DLC does not inhibit protein synthesis by showing no change when CHX is used in combination with DLC or when CHX is used alone (Fig 2E and 2F). Meanwhile, DLC had no effect on the changes in mRNA levels caused by SVA infection as determined by qPCR(Fig 2G). These results indicate that, DLC could inhibit the protein degradation of LAMP1 induced by SVA.

We found that DLC restored lysosomal damage caused by SVA infection. Lysotracker was used to check the effect of DLC on the number of lysosomes. We found that DLC could inhibit the reduction of the number of lysosomes after SVA infection and maintain lysosomes in normal physiological state (Fig 2H and 2I). SVA infection could lead to lysosomal membrane rupture and result in an increase in lysosomal membrane permeability when detected by acridine orange staining. We found that the lysosomal membrane rupture was alleviated by addition of DLC, suggesting that DLC played a role in protecting the integrity of the lysosomal membrane(Fig 2J and 2K). Next, we examined the changes in VP2 protein expression levels under lysophagy or overexpression and knockdown of LAMP1. Induction of lysophagy promoted SVA production, while overexpression or knockdown of LAMP1 did not enhance SVA replication (Fig 2L-O).

**Fig 3:**
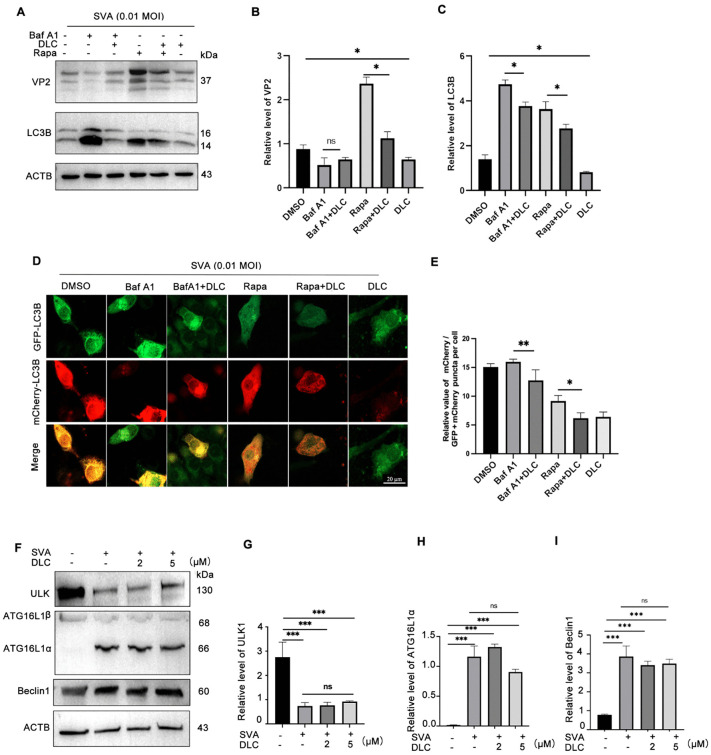
DLC reduced the autophagic flux that was elevated by SVA infection without blocking autophagy. **(A-C)** The expression levels of VP2 and LC3B proteins **(D,E)** GFP-mCherry-LC3B plasmid was transfected into BHK-21 cells 12 h later, the transfected cells were infected with the autophagy inhibitor Baf A1(5 μM) and the autophagy activator Rapamycin (5 μM) and DLC (5 μM) respectively or in combination. The autophagy flow was detected by confocal microscopy.The ratio of mCherry spots to mCherry+GFP spots in at least 10 individual cells was analyzed and statistically analyzed (n > 10). **(F-H)** The cells were infected with SVA at a MOI of 0.01 and treated with different concentrations of DLC (0,2,5 μm) for 24 hpi. The cell lysates were collected to detect the protein expressions of ULK1, ATG16L and Beclin1 in the autophagy pathway. Scale bar: 20 μm. (*p < 0.05, **p < 0.01, ***p < 0.001, n = 3).

To further determine whether DLC reduces SVA production in host cells by protecting lysosomes and inhibiting lysophagy, we used BafA1, Rapamycin and DLC separately and in combination within 24 hours of viral infection, and examined the expression level of LC3B, a regularized marker of autophagy, and the expression level of SVA VP2 protein. BafA1 is an autophagy inhibitor that inhibits lysosomal acidification. Treatment of BafA1 reduced protein levels of VP2 protein compared to the infected group (Fig 3A and 3B). We speculate that this extensive disruption of BafA1 most likely interferes with processes that depend on normal endosome-lysosomal acidification at earlier stages of the viral life cycle, resulting in overall inhibition of replication efficiency.However, the expression level of VP2 increased after the addition of rapamycin to activate autophagy, and the increase of VP2 was significantly inhibited when combined with DLC (Fig 3B). We also found that DLC could inhibit the formation of autophagosomes and reduces the autophagy flux by examining the expression level of LC3B (Fig 3C). To detect the changes of autophagy flux under DLC intervention, we transfected GFP-mCherry-LC3B into BHK-21 cells. This dual-fluorescent plasmid could monitor autophagic flow, and only red fluorescence could be detected when GFP fluorescence was quenched after fusion of autophagosomes and lysosomes, while yellow fluorescence could be detected when autophagy was inhibited.DLC inhibited the increase in yellow spot aggregation compared to Baf A1 alone and also inhibited the red aggregation induced by Rapa, suggestting that DLC acts by inhibiting the formation of autophagic vesicles (early autophagy) rather than blocking the fusion of autophagosomes with lysosomes (Fig 3D and 3E).Taken together these results indicate that the proliferation of SVA can lead to lysosomal damage and trigger lysophagy, while DLC attenuates the effects of SVA by protecting the lysosomes.ULK1, a central component of the autophagy mechanism, activates autophagy and induces ATGL161α to form a multimercomplex, which determines the formation of mature autophagosomes [28].The results showed that the expression of ULK1 was decreased, while ATG16L1α were significantly increased after SVA infection. However, the protein levels in this pathway were not inhibited by DLC treatment compared with the control group (Fig 3F-H). These results indicated that DLC could control lysophagy caused by lysosomal membrane damage during SVA infection, protect the fusion of damaged lysosomes with autophagic vesicles, but did not inhibit autophagic flow.

### DLC inhibited the degradation of LAMP1 through the ubiquitin-proteasome pathway and attenuated the interaction between Gal3 and TRIM16

**Fig 4:**
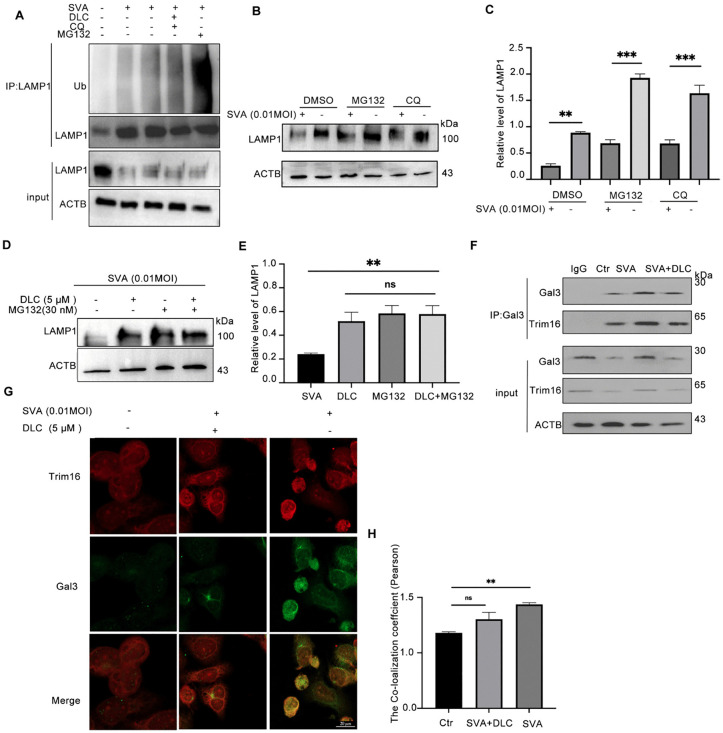
DLC reduces ubiquitin-proteasome degradation of LAMP1 induced by SVA infection and inhibits lysophagy. **(A)** The ubiquitination level of LAMP1 treated with CQ (10 μM) and MG132 (50 nM) before and after SVA infection. **(B,C)** Effect of CQ (50 μM) and MG132 (50 nM) on LAMP1 protein levels before and after SVA infection. **(D,E)** Effects of DLC alone or in combination with MG132 (50 nM) on LAMP1 protein levels after SVA infection. **(F)** Effect of DLC on the interaction between endogenous TRIM16 and Gal3 in SVA infected cells by Co-IP (After Gal protein was immunoprecipitated, Gal3 and TRIM16 proteins were respectively used for detection). **(G,H)** Colocalization changes of TRIM16 and Gal3 in cells with and without DLC (5 μM) after SVA infection (0.01MOI, 24 hpi) and quantitative analysis.(*p < 0.05, **p < 0.01, ***p < 0.001, n = 3).

We hypothesized that the degradation of LAMP1 protein is due to the damage of lysosomal membrane caused by SVA infection and will be degraded through the selective autophagy pathway. Chloroquine (CQ) is known to inhibit lysosomal activity and autophagy while MG132 is a potent proteasome inhibitor. To understand the mechanism of action of DLC, cells were treated with DLC alone or combined with CQ or MG132 to detect the degradation of LAMP1. The results showed that the combination of CQ, M132 and DLC could accumulate the ubiquitin labeled LAMP1 (Fig 4A), but by comparing the LAMP1 levels in Input, we can see that the combination of CQ and DLC caused a significant difference in LAMP1 protein levels, while the combination of DLC and MG132 caused a significant difference in LAMP1 protein levels. The levels of LAMP1 in Input were generally consistent, indicating that DLC had a synergistic effect with MG132 in inhibiting the degradation of LAMP1, that is, DLC inhibition inhibited the degradation of LAMP1 after SVA infection through the ubiquitin-proteasome pathway (Fig 4A). Although we found by Western Blot that CQ and MG132 were added before and after virus infection, both of them could restore the protein level of LAMP1 (Fig 4B and 4C), indicating that LAMP1 could be degraded not only through the ubiquitin-proteasome pathway, but also through the autophagy-lysosomal pathway. However, further experiments showed that the protein levels of LAMP1 in each group were not significantly different after treatment with DLC alone or in combination with MG132, and were significantly higher than those in the SVA infection group, indicating that DLC indeed inhibited the degradation of LAMP1 by participating in the inhibition of the ubiquitin-proteasome pathway (Fig 4D and 4E).These results suggest that DLC plays the same role as MG132 in inhibiting the physiological process of LAMP1 degradation.The relationship between the autophagy-lysosomal pathway and the ubiquitin-proteasome pathway in the degradation of LAMP1 after infection, combined with the results of this study, can clearly show that the two are independent events. DLC mainly exerts its effect by inhibiting the ubiquitin-proteasome pathway.

During lysophagy, Gal3 tags the damaged lysosomes and recruits the E3 ubiquitin ligase TRIM16 to degrade LAMP1 [29]. Therefore, we examined the binding of Gal3 to TRIM16 after DLC treatment. We found that DLC reduced the colocalization of Gal3 and TRIM16. The decreased interaction between Gal3 and TRIM16 was confirmed by Co-IP (Fig 4F). Furthermore, the changes in co-localization of Gal3 and TRIM16 also reflect the intervention of DLC on lysophagy after SVA infection (Fig 4G and 4H).Taken together, DLC inhibited the degradation of LAMP1 through the ubiquitin-proteasome pathway, blocked the interaction between Gal3 and TRIM16, and reduced lysophagy after SVA infection.

### DLC directly binds to LAMP1 and inhibits lysophagy by maintaining LAMP1 stability

**Fig 5:**
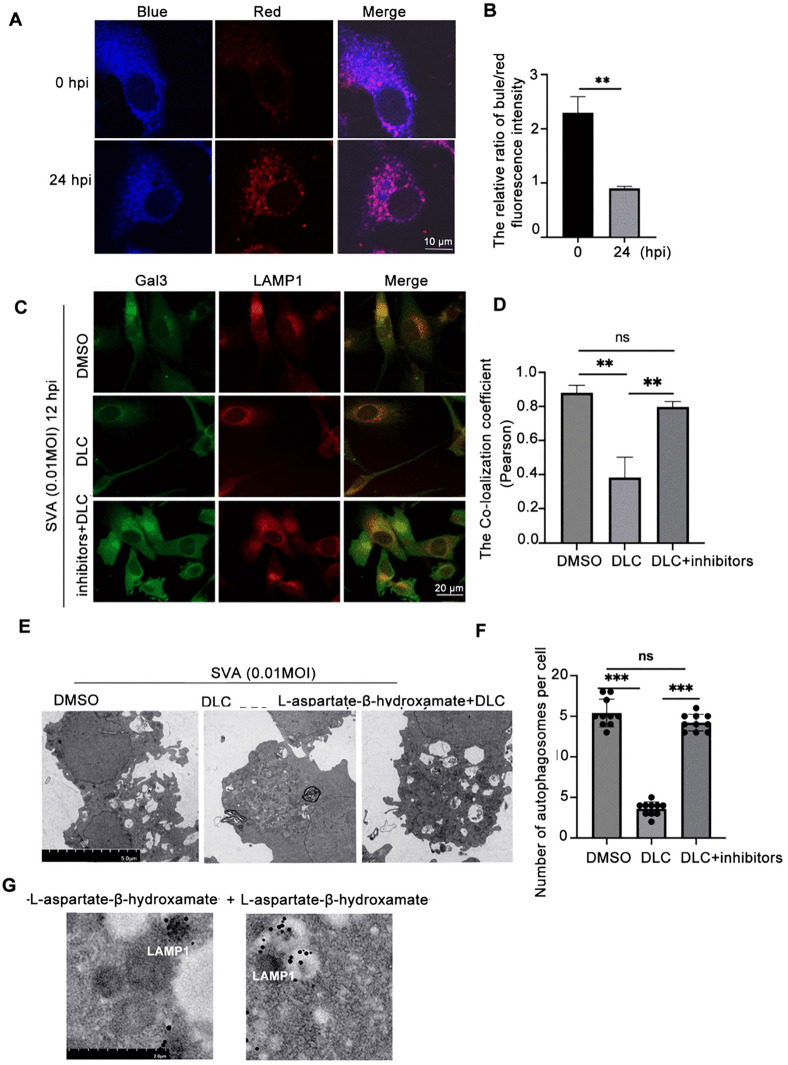
DLC works by binding endogenous sulfur dioxide. **(A,** **B)** After 0 and 24 h of SVA infection, probe CIJ (5 μM) for the detection of endogenous sulfur dioxide was added, Images were obtained at 420-600 nm for blue fluorescence and 600-700 nm for red fluorescence, respectively, and the relative fluorescence intensity ratios of blue/red for individual cells in three random fields were selected and counted. **(C, D)** Changes in colocalization of LAMP1 and Gal3 cells in response to SVA infection (0.01MIOI, 12hpi) and DLC (5 μM) treatment were examined by confocal microscopy after inhibition of endogenous SO_2_ production, Through statistical analysis of the Pearson’s coefficients of the co-localization of the two in three random fields of view.Scale bar: 20 μm. **(E,F)** The number of autophagosomes in the above groups was observed by electron microscopy, and the number of autophagosomes in 10 randomly selected cells was counted and counted, Scale bar:5 μm (*p < 0.05, **p < 0.01, ***p < 0.001, n = 3).

Next, we further explored the mechanisms by which DLC works. DLC can recruit intracellular free SO_2_ to localize on specific proteins and play a role in post-translational modification [24]. To investigate whether DLC plays a role by regulating intracellular SO_2_ after SVA infection, we used a SO_2_ fluorescent probe CIJ to detect the intracellular SO_2_ level after SVA infection [30]. At 0 and 24 hours after SVA infection, cells were incubated with CIJ (5 μM) for 1 hour and the blue and red fluorescence of the cells were observed under 546 nm excitation light by confocal microscopy. The concentration of SO_2_ was determined by the fluorescence intensity of the blue/red channels. We found that the concentration of SO_2_ within cells decreased 24 hours after SVA infection (Fig 5A and 5B). Inhibiting or activating the activity of AAT is an important way to regulate endogenous SO_2_ level. The commonly used AAT inhibitor L-Aspartic acid β-hydroxamate can reduce endogenous SO_2_ content [31–33]. We next examined the protective effect of DLC on lysosomes after SVA infection when treated with the addition of L-Aspartic acid β-hydroxamate.The results showed that at the same dose and time of SVA infection (0.01MOI, 12hpi), the co-localization of Gal3 and LAMP1 in DLC-treated cells was decreased, as indicated by the decreased green fluorescence representing Gal3 and the decreased yellow fluorescence after co-localization of LAMP1 and Gal3. However, when inhibitors were added to inhibit the production of SO_2_, the colocalization of the two was not significantly different from the intensity after SVA infection(Fig 5C and 5D). The number of autophagosomes in 10 randomly selected cells was counted. The results showed that the inhibition effect of DLC on autophagy was reduced after the combination of DLCS and inhibitors, and the number of autophagosomes was not significantly different from that after SVA infection (Fig 5E and 5F).Thus, we demonstrated that DLC inhibits the activation of lysosomal autophagy by SVA infection with the involvement of endogenous SO_2_.

**Fig 6:**
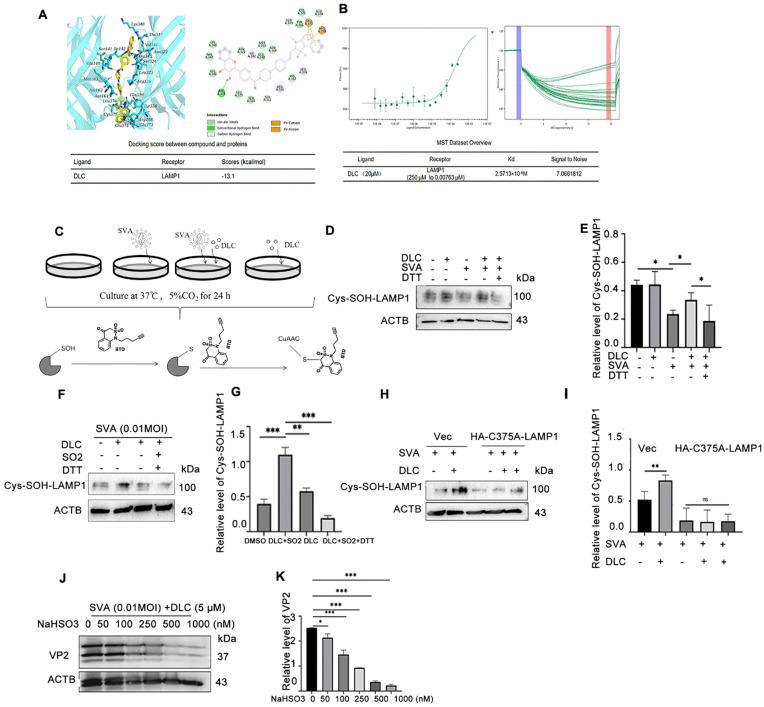
DLC directly binds to LAMP1 and regulates the sulfenylation of LAMP1 through SO_2_ to reduce the production of SVA. **(A)** Predicted structure of LAMP1(blue) and DLC (yellow). The binding sites of DLC on LAMP1 protein were predicted by Docking (Glu140, His162, Met163, Asn164, Asn322, Ser324, Ile325, Tyr336, Val346, Thr347, Lys348, Cys375, Glu374) **(B)** The molecular binding assay through MST experiments (Signal to Noise Ratio>4). **(C)** The sulfenylation labeling of proteins in cells and the flow diagram of immunoblotting.(D,E) After different treatments of BHK-21 cells, cells were harvested according to the procedure shown in **(D-E)**, the thiolation level of LAMP1 protein was measured, and the reversibility of this oxidation was confirmed by DTT (10mM). **(F,G)** The sulfonylation of LAMP1 by DLC was detected by adding SO_2_ donor NaHSO_3_ (500 nM); - **(H,I)** Sulfenylation level of Cys375A-LAMP1 after SVA infection and DLC treatment. **(J,K)** The expression of VP2 in SVA infected BHK-21 cells treated with different concentrations of SO_2_ donor NaHSO_3_. (*p < 0.05, **p < 0.01, ***p < 0.001, n = 3).

To investigate how DLC functions on LAMP1 protein, we used bioinformatics analysis to predict the binding of DLC to LAMP1 by Molecular Docking. The DLC and LAMP1 binding sites form stereoscopic complements (Fig 6A). Specifically, the oxygen atom of DLC interacts with Arg345 in LAMP1 to form a hydrogen bond; The two carbon atoms of DLC interacted with Ser141 and Gln329 in LAMP1 to form two C-H bonds. The benzene rings of DLC interacted with Arg298 and Gln329 in LAMP1 to form two C-H bonds, respectively. DLC forms hydrophobicity with residues Ile142, Arg326, and Leu376. DLC could also interact with the His162, Ser324, Ile325, Val346, Thr347, Lys348, Asn322, Glu140, Met163, Asn164 and Tyr336 sites of LAMP1. A VDW interaction is formed between Cys375 and Glu374 residues.The interaction between DLC and LAMP1 was further confirmed by MST experiments. The binding dissociation constant Kd of DLC and LAMP1 was 2.5713 × 10^-6^M, and the Signal to Noise was 7.0661812 (Fig 6B). In conclusion, DLC can exert its effects by directly binding to LAMP1.

### DLC enhances sulfenylation modification of LAMP1 to inhibit LAMP1 ubiquitination degradation after SVA infection

It has been shown that sulfenylation modification on cysteine can inhibit ubiquitination and maintain protein stability [34,35]. Since that DLC is a sulfur dioxide probe and can bind to Cys375 of LAMP1, it is reasonable to assume that DLC may inhibit LAMP1 degradation by increasing the sulfenylation modification of LAMP1. We captured intracellular sulfenylated proteins with a BTD probe and quantified proteins using biotin-conjugated western blot (Fig 6C). The feasibility of the BTD probe to capture the sulphonylated modified proteins was verified (S5 Fig). We found that the sulfenylation level of LAMP1 was decreased after SVA infection, while the thiolation level of LAMP1 was significantly increased after DLC treatment, and this modification could be reduced by DTT(10 mM) (Fig 6D and 6E).Since sulfenylation requires the involvement of reactive sulfur in the cell, to further verify the occurrence of sulfenylation, we utilized sodium bisulfite as an exogenous sulfur dioxide generator to elevate the intracellular SO_2_ level and detected the changes in the sulfenylation level of LAMP1 during this process. The results showed that the sulfenylation level of LAMP1 increased in correlation with the concentration of sodium bisulfite (Fig 6F,G). To determine whether Cys375 on LAMP1 serves as a site for sulfenylation, we constructed a mutant of LAMP1 (C375A) and examined the sulfenylation levels after DLC treatment. DLC could only enhance the sulfenylation of wild-type LAMP1 but not the Cys375 mutant (Fig 6H and 6I). We also verified that the expression of VP2 decreased significantly with the increase of SO_2_ concentration when treated with DLC (Fig 6J and 6K). These results demonstrated that DLC increased SO_2_-regulated sulfenylation in cells and inhibited SVA production by increasing the sulfenylation level of LAMP1 at Cys375.

Since DLC can bind intracellular SO_2_ and act on cysteine through post-translational modification, we hypothesized that DLC inhibits the degradation of LAMP1 probably by antagonizing the cysteine ubiquitination on LAMP1. Therefore, we analyzed the effect of DLC on the ubiquitination of LAMP1 cysteines. We replaced nine cysteines on LAMP1 with alanine, and transfected them into HEK-293T cells to detect changes in ubiquitination levels between WT-LAMP1 and these mutants after viral infection. Interestingly, we observed that SVA infection elevated the ubiquitination level of transfected WT-LAMP1, which could be significantly suppressed by DLC treatment. However, after the C375A transfected, the ubiquitination level of LAMP1 caused by SVA infection was down-regulated and DLC was no longer able to exert its effect (S6A-S6D Fig). In contrast, mutations at positions 25, 41, 80, 155, and 191, were still able to show a decrease in ubiquitination levels after DLC treatment, whereas the ubiquitination at positions 231, 269, and 338 was not significantly different from that of the wild type, and none of them showed a trend similar to that observed in C375A mutant. We further analyzed the ubiquitination sites of LAMP1 after SVA virus infection using LC-MS/MS.The results showed that ubiquitination on LAMP1 Cys375 was detected in BHK-21, PK-15 and HEK-293t cells infected with SVA (S6E-S6G Fig).The total ion current chromatograms of the samples are shown in S7 Fig.The ubiquitination sites of 191, 231, 338 and 375 cysteine could be detected (S8 Fig), which proved that viral infection did indeed cause the ubiquitination of LAMP1 cysteine.These results suggest that Cys375 is a cysteine ubiquitination site on LAMP1 and that DLC can promote sulfenylation of LAMP1 at the same site, therefore inhibit cysteine ubiquitination and degradation of LAMP1 after SVA infection.

### DLC reduces the replication of SVA in BHK-21cell and PK-15 cell

**Fig 7:**
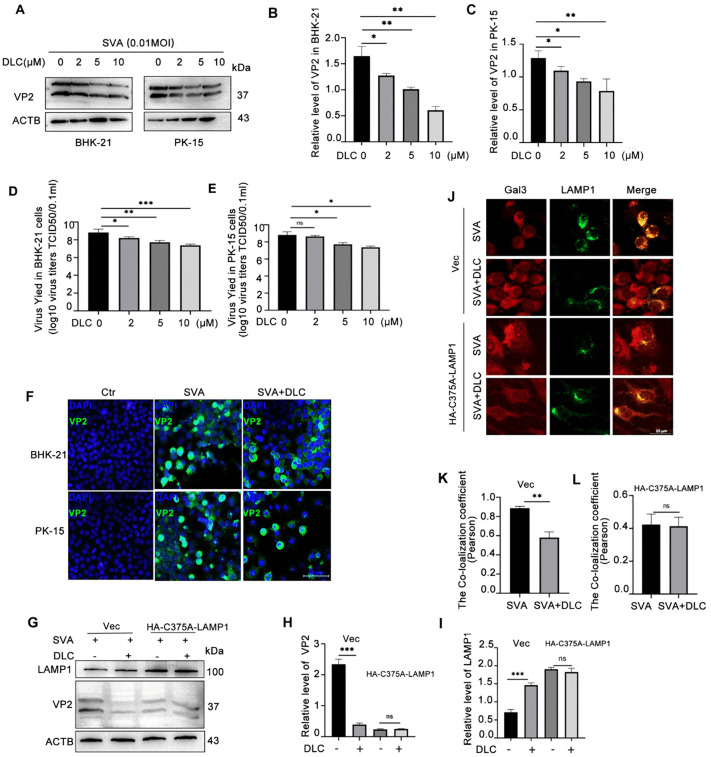
DLC reduces the production of SVA virus in BHK-21 and PK-15 cells. **(A-C)** BHK-21 cells and PK-15 cells were pretreated with 0,2,5,10 μM DLC for 30 min before virus infection, and the virus venom was added according to 0.01 MOI. Protein samples were collected and the expression level of virus VP2 in the cells was detected by western blot assay. **(D,E)** The effects of DLC on SVA virus titer in BHK-21 cells and PK-15 cells were detected by TCID_50_ method. **(F)** Immunofluorescence assay was used to observe the number of VP2-positive cells in BHK-21 and PK-15 cells after SVA infection and DLC treatment. Scale bar: 50 μm **(G-I)** After transfection of Vec and HA-C375A-LAMP1 in BHK-21 cells, DLC was added to detect the protein levels of LAMP1 and VP2. **(J-L)**After transfection of Vec and HA-C375A-LAMP1 in BHK-21 cells, the colocalization changes of Gal3 and LAMP1 were detected under confocal microscopy.Scale bar: 20 μm. (*p < 0.05, **p < 0.01, ***p < 0.001, n = 3).

The above results revealed that SVA can complete its replication by inducing lysosomal membrane damage and autophagy, while DLC can act as an inhibitor of lysophagy by preventing the degradation of LAMP1 and ultimately reduce the production of SVA. These data indicates that DLC can be used as a small molecule drug against SVA virus. Therefore, we examined the inhibitory effect of DLC on SVA in BHK-21 and PK-15 cell lines. Due to the large amount of virus replication in the host cells will lead to cell round, necrosis, exfoliation and other cytopathic effect (CPE), after adding DLC treatment, we observed under the microscope that DLC had a significant inhibitory effect on CPE caused by SVA (S9 Fig). Next, we found that DLC could reduce the production of SVA and the copy number of the virus (Fig 7A, 7B and 7C).The effects of different concentrations of DLC on intracellular virus titers indicated that DLC reduced the replication ability of SVA (Fig 7D and 7E). Cytological analysis of viral structural protein VP2 revealed that DLC could reduce the number of VP2 positive cells after SVA infection, and the number of positive cells was decreased from 100% to 42%-50%(Fig 7F).mRNA was extracted and the expression level of VP2 was detected by Real-time fluorescent qPCR (S10 Fig). Meanwhile, we also found that the expression of VP2 protein was decreased in the cells transfected with C375A-LAMP1, and there was no significant difference observed before and after DLC treatment (Fig 7G-I).In addition, the colocalization coefficient of LAMP1 and Gal3 in transfected C375A-LAMP1 cells was less than 0.5, indicating that the level of lysosomal autophagy was reduced, and there was no statistically significant difference before and after DLC treatment (Fig 7J-L). These results demonstrated that DLC could inhibit SVA replication and lysosomal autophagy by regulating the sulfenylation of LAMP1 Cys375 and inhibiting ubiquitination.

### DLC reduced the proliferation of SVA and alleviated organ damage in BALB/c mice

**Fig 8:**
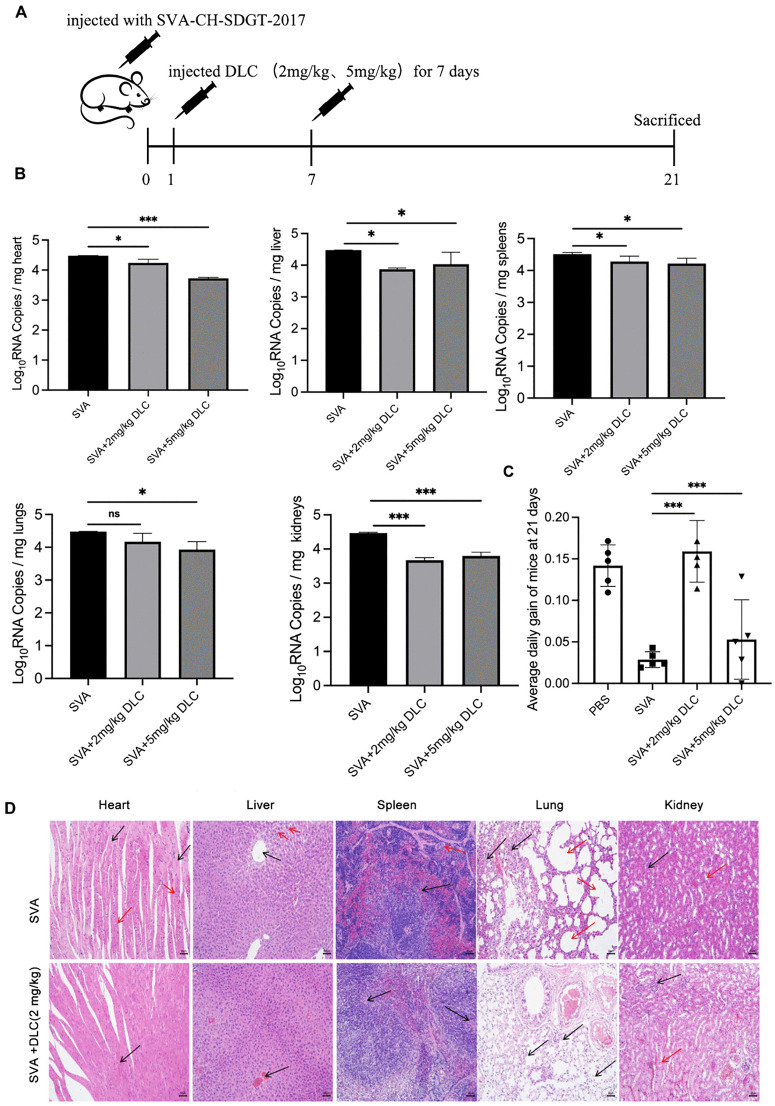
DLC reduced the proliferation of SVA on mice and the damage to organs. **(A)** Experimental procedure of SVA injection and DLC injection in mice. **(B)** Determination of SVA viral load (Log_10_ RNA Copies/ mg)in various organs of mice in different groups by qPCR. (*p < 0.05, **p < 0.01, ***p < 0.001, n = 5) **(C)** 21-day average daily gain of mice in different groups. (*p < 0.05, **p < 0.01, ***p < 0.001, n = 5). **(D)** Pathological changes of various organs after virus infection and DLC treatment observed by HE staining.

**Fig 9:**
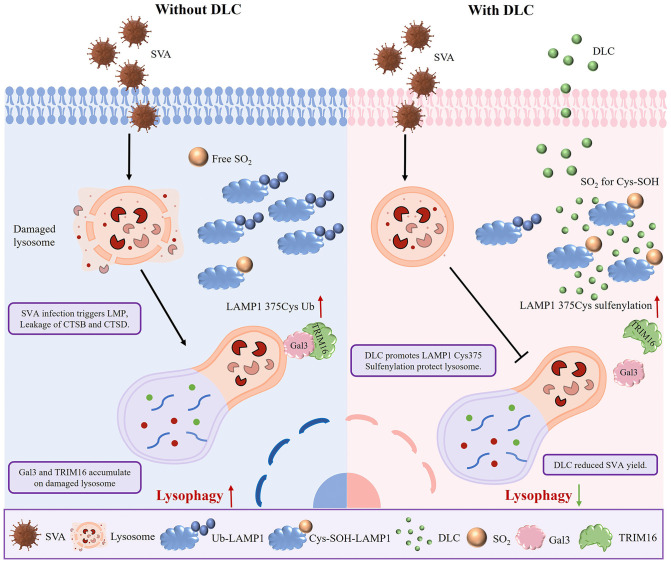
Schematic diagram of DLC promoting sulfinylation of LAMP1 and inhibiting lysophagy induced by SVA infection. In the absence of DLC, SVA infection caused lysosomal damage, increased LAMP1 protein ubiquitination and decreased SO_2_ levels. In the presence of DLC, the SO_2_ level is increased, and DLC binds it and regulates its function. By binding to LAMP1, DLC increases the sulfation of 375 cysteine and reduces lysosomal damage caused by SVA infection. Lysophagy is inhibited, thereby inhibited lysosomal damage.

To verify the antiviral effect of DLC *in vivo*, we established a BALB/c mouse SVA infection model and treated SVA-infected mice with intraperitoneal injection of DLC (Fig 9A). Measurement of the viral load in the organs of mice showed that compared with the mice in the virus-infected group, the injection of DLC could reduce the viral load in the organs, indicating that DLC could inhibit the replication of SVA *in vivo* (Fig 8B). We also observed that there was a significant difference in average daily weight gain over 21 days in mice given low or high doses of the drug. The weight growth trend of mice in low dose group was higher than that in healthy control group (Fig 8C). Through the pathological observation of the injury and inflammation degree of different organs of mice in different two groups, we found that in the group without DLC treatment, mice showed different degrees of tissue morphological changes and slight inflammatory cell infiltration in different organs, especially in the spleen and lung (Fig 8D). The overall structure of myocardial tissue in the virus infection group was abnormal, which was manifested as localized degeneration and necrosis of myocardial fibers, nuclear atrophy, and loose structure destruction (black arrow). There was mild inflammatory cell infiltration in the myocardial interstitium. The overall structure of liver tissue was slightly abnormal, and the lobular structure was basically preserved. The hepatic cord cells were arranged in a stellate -radial pattern around the central veins. Some hepatocytes showed mild steatosis with small lipid droplets in the cytoplasm. There was no obvious inflammatory cell infiltration in the interstitial tissue. The overall structure of the spleen was abnormal, the edge band of the lymphatic sheath of the white pulp was broken and arranged disorderly, and the boundary between the white pulp and the red pulp was blurred (black arrow). Of note, the proliferation of lymphocytes and macrophages in the red medulla was evident (red arrows). The overall structure of the lung tissue was abnormal, with destruction or absence of local alveolar structure and mild interstitial inflammatory cell infiltration (black arrows). In addition, localized alveolar-septal thickening and compensatory emphysema (red arrows) were observed. The tissues of mice treated with DLC (2 mg/kg) maintained normal structure, and no obvious inflammatory cell infiltration was observed in the interstitium(Fig 8D). The expression of SVA VP2 protein in various organs of SVA infected and DLC-treated mice was also examined by immunohistochemical staining (S12 Fig). Statistical analysis of the mean optical density values of VP2-positive cells showed that the content of VP2 in each organ of the DLC-treated mice was lower than that of the SVA infected group. Among them, the heart, liver, kidney and lung had extremely significant differences, which were also basically corresponding to the pathological results after H&E staining. In general, after DLC treatment, the viral load of infected mice decreased, and the organs maintained normal physiological morphology without obvious inflammatory cells. Based on the above experiments, we demonstrated the inhibitory ability of DLC on SVA at the *in vivo* level, and further clarified the potential of DLC as a candidate drug for anti-SVA.

## Discussion

In this study, we demonstrated that SVA causes lysosomal damage and triggers lysophagy, and that autophagy is required for virus replication. Lysosomes play a crucial role in the cellular degradation processes, including the breakdown of viral particles after endocytosis. The endocytic pathway is essential for virus entry into host cells. This pathway typically involves viral transport through endosomes and lysosomes, where an acidic environment can facilitate viral uncoating and release of the viral genome into the cytoplasm [36]. In the case of porcine sapelovirus (PSV), which is closely related to SVA, studies have shown that its entry into porcine small intestinal epithelial cells (IPEC-J2) is dependent on specific endocytic pathways, including caveolae/lipid raft-mediated endocytosis. This process is pH-dependent and requires dynamin and phosphoinositide 3-kinase (PI3K), but is independent of clathrin and macropinocytosis, indicating that lysosomal pathways are involved in the viral life cycle [37]. Moreover, the interaction of viruses with lysosomes can also influence their replication dynamics. It has been demonstrated that the porcine reproductive and respiratory syndrome virus (PRRSV) utilizes low pH-dependent endosomal pathways for membrane fusion and entry into host cells. The involvement of lysosomal enzymes, such as cathepsins, in the processing of viral proteins during entry has been verified [38].

Here we showed that SVA infection disrupts the permeability of the lysosomal membrane, allowing the flow of acid hydrolases to the cytoplasm, leading to damaged lysophagy during its replication. It should be noted that while this study primarily focuses on lysosomal damage and lysophagy as a key mechanism in SVA infection, we recognize that viral pathogens often interfere with multiple cellular compartments and pathways. Lysosomal impairment may not be the initial triggering event but rather a subsequent effect within a broader network of virus-host interactions. For instance, various viruses are known to induce mitochondrial stress, endoplasmic reticulum (ER) disruption, or inflammatory signaling cascades, any of which could independently or synergistically contribute to lysosomal damage and overall cellular dysregulation [39,40].Future studies that delve into the temporal sequence and hierarchical relationships among these pathways will be crucial for comprehensively elucidating the mechanistic landscape of SVA infection. Furthermore, this study suggests that SVA-induced lysosomal damage can trigger a host lysophagic response to remove compromised lysosomes. However, from the viral perspective, prolonged retention within lysosomes increases the risk of degradation and reduces infectivity. Therefore, it is likely that SVA employs certain mechanisms to rapidly escape into the cytoplasm before lysophagic clearance occurs. In addition, we also examined the expression of LAMP2 and LAMP3 in (S2 Fig). Since LAMP2 is a key factor in the chaperon-mediated autophagy (CMA) pathway, its dynamic changes may significantly affect lysosomal stability, autophagic clearance, and viral release kinetics.As a stress-induced member of the LAMP family, LAMP3 is transcriptionally activated during lysosomal injury and participates in membrane repair and vesicle transport. Regarding the reason why the overall lysosomal dysfunction caused by SVA directly triggers the specific upregulation of LAMP3, we speculate that SVA may utilize a response similar to that of the influenza A virus hijacking LAMP3-positive vesicles for nucleoprotein transport and viral particle assembly to support its own replication, converting the stress compensation of cells into a previral mechanism. Future studies using LAMP2A knockdown or knockout models will provide important insights into our understanding of the functional interplay between CMA and SVA infection. Suggesting our understanding of the temporal coordination among lysosomal penetration, viral release, and autophagy activation will provide important insights into the identification of potential therapeutic targets.

Based on intramolecular charge transfer (ICT) and Foster resonance energy transfer (FRET) platforms, DLC uses the flavone moiety and benzindole derivatives as raw materials to react with SO_2_ derivatives, which can image exogenous and endogenous SO_2_ derivatives in HeLa, HepG2 and LO2 cells. It has good biocompatibility and photostability [23].In our previous study, we found that DLC inhibited HUVECs senescence induced by high glucose by promoting the decomposition of lipid droplets and protecting V-ATPase channels on lysosomes [24]. After lysosomal membrane rupture, the intensity of red fluorescence in the LMP assay was positively correlated with the degree of penetration of acidic components. We found that the acidity of lysosomes decreased after SVA infection, while the acidic environment inside lysosomes could be restored after DLC treatment, which was similar to our previous results. The specific mechanism needs to be further explored.Meanwhile, the results in Fig 3F demonstrate that DLC does not affect the regulation of ULK-axis-related proteins. It can be seen from this that once lysosomal autophagy caused by SVA infection occurs, the related proteins on the ULK axis will change. Importantly, DLC only maintains the stability of a part of the LAMP1 protein through post-translational modification of sulfur dioxide. DLC does not target or regulate other components of the autophagy pathway, including the ULK1 axis (ULK1, Atg16L) or key proteins in downstream autophagy mechanisms. This explains why DLC has only a limited effect on overall autophagy.In addition, we found that Rapamycin and LLOME could increase the expression of VP2, indicating that lysophagy was involved in the replication and proliferation of SVA. It should be noted that chronic inhibition of lysophagy by DLC may lead to the accumulation of undegraded, modified LAMP1, which could disrupt lysosomal homeostasis and reformation over time. While our findings demonstrate a net beneficial effect of DLC in the context of acute viral infection, potential long-term detrimental effects should be carefully considered for therapeutic applications.

Previous studies of LAMP1 have often focused on glycosylation sites. Glycosylation of LAMP1 involves the addition of a carbohydrate moiety to the protein, which is essential for its proper folding and transport to the lysosomal membrane. Alterations in the glycosylation pattern of LAMP1 are associated with various pathological conditions. In addition to glycosylation, the phosphorylation status of LAMP1 may affect its ability to interact with other proteins involved in lysosomal transport and fusion processes [41].The dynamic nature of post-translational modifications of LAMP1 suggests that they may play a role in the cellular response to stress or changes in the cellular environment. For example, under oxidative stress conditions, modification of LAMP1 may affect its function in autophagy and lysosomal degradation pathways, which are essential for cellular homeostasis and removal of damaged organelles and proteins [42]. During PEDV infection, LAMP1 expression was observed to be downregulated at specific time points post-infection, suggesting that the virus may inhibit lysosomal fusion to promote its replication.This inhibition of autolysosome formation is beneficial for PEDV, because it allows the virus to escape degradation and improve its replication efficiency. Moreover, knockdown of LAMP1 increases PEDV protein expression and viral replication, further supporting the notion that LAMP1 plays a negative regulatory role in viral replication [43]. Here we found that the post-translational modification of LAMP1 cysteine is also critical. SVA infection can lead to the ubiquitination and degradation of LAMP1 and damage the structural integrity of the lysosomal membrane, while DLC can bind to LAMP1 Cys375 and increase the sulfenylation modification to prevent ubiquitination degradation of LAMP1. Recent studies have highlighted a potential link between sulfenylation and ubiquitination: sulfenylation can affect the ubiquitination process by modifying specific cysteine residues in ubiquitin ligases or substrates, thereby affecting their activity or stability [34]. This is particularly important in the context of oxidative stress, where elevated levels of reactive oxygen species can lead to increased protein sulfenylation, potentially affecting their ubiquitination status [44].Therefore, our study uncovered the crosstalk between these two modifications caused by viral infection.

Although the specific viral proteins mediating SVA-induced lysosomal damage remain to be fully elucidated, several reasonable mechanisms can be proposed based on the known examples of small RNA viruses. SVA infection has been proven to lead to a significant decrease in LAMP1 levels accompanied by an increase in lysosomal membrane permeability, suggesting that the virus may directly damage the lysosomal membrane structure [25]. In addition, the endolysosomal pathway transport of SVA within cells and the establishment of viral replication complexes in the intimal system may cause physical interference, affecting lysosomal structure and membrane transport processes [45–47]. Virus-encoded proteases (such as 2A or 3C) may disrupt lysosomal integrity by directly cleaving lysosomal membrane proteins or key host factors that regulate autophagy. Among them, SVA 3C has been reported to cleave various selective autophagy receptors or autophagy-related factors, such as OPTN and DCP1A, thereby altering the fluidity of the autophagy-lysosomal pathway [48,49]. Another possibility is that the 2B protein encoded by SVA is similar to viroporin of other small RNA viruses, which can form pores on the inner membrane of cells, causing lysosomal membrane permeation, loss of ion gradient and leakage of contents [50,51]. These potential mechanisms are all consistent with the phenotypes of LAMP1 decline and increased lysosomal membrane permeability that we observed, and together they constitute the possible pathways by which the virus triggers lysosomal autophagy. Clarifying the precise effector molecules of SVA in destroying lysosomes will be an important direction for future research.

Sulfenylation modification has attracted attention in various fields including medicinal chemistry and antiviral research. Recent studies have highlighted the potential of sulfinyl compounds to target viral proteins and pathways [52,53]. Synthesized sulforanylated derivatives exhibit potent inhibitory activity against SARS-CoV-2 Mpro, demonstrating the potential of sulfenylation to enhance antiviral activity [54].In addition, the introduction of a sulfur group can affect the pharmacological properties of the compound, the ability of sulfonated flavonoids to inhibit viral replication. DLC is a SO_2_ probe synthesized based on the flavonoid derivative as its donor. Here we verified that DLC has a good antiviral effect on SVA at the cellular level and *in vivo* level, which also suggests that sulfenylation modification is of great significance for the development of broad-spectrum antiviral drugs. However, in in vivo experiments, we observed a significant inhibition of weight gain in the high-dose group. This phenomenon is consistent with the preclinical and clinical reports of various antiviral drugs. It is considered that its mechanism mainly involves over-activation of the target and off-target effects. For instance, at high doses, the HIV protease inhibitor lopinavir inhibits the mTOR pathway [55], In addition, it may also mediate metabolic damage due to off-target effects, typically characterized by inhibition of mitochondrial DNA polymerase γ by nucleoside analogues at high doses, inducing ATP synthesis disorder and muscle wasting [56].We agree that inhibiting lysophagy may impair long-term lysosomal function due to the accumulation of undegraded material.Our data indicate that lysophagy inhibition confers a beneficial effect following SVA infection in the short term; however, prolonged inhibition could disrupt normal lysosomal function.

Therefore, lysophagy inhibitors may be more suitable in acute settings—such as infection or injury.Future studies should aim to define the optimal therapeutic window for lysophagy inhibition and further validate these effects in aging and other relevant models.In conclusion, the use of dynamic metabolic monitoring of multiple organs to define the dose-effect critical point will provide a safe guarantee for clinical translation in the future.In summary, our study provides new insights into the regulatory mechanisms of lysophagy during SVA infection (Fig 9). By modulating LAMP1 sulfenylation and ubiquitination, DLC holds promise as a therapeutic candidate for antiviral drug development.

## Materials and methods

### Ethics statement

The animal experiments in this thesis were strictly in accordance with the guidelines of ARRIVE (Animal Research: Reporting of In Vivo Experiments) and approved by the Animal Ethics Committee of Shandong Academy of Agricultural Sciences (IACUC approval number: TASYM-2024–036).

### Cells, viruses and reagents

BHK-21 cells, PK-15 cells and HEK-293T cells were grown at 37°C with 5% CO_2_ in Dulbecco’s modified Eagle’s medium (DMEM; Invitrogen 1917773) containing 10% fetal bovine serum (FBS; Invitrogen, 10099141C). SVA-CH-SDGT-2017 strain was isolated by Shandong Provincial Key Laboratory of Animal Disease Control & Breeding, Institute of Animal Science and Veterinary Medicine Shandong Academy of Agricultural Sciences. The rabbit monoclonal antibody specific to SVA VP2 was prepared in our laboratory and has been reported previously. The antibody of ACTB (122M4782) was from Sigma Aldrich (St. Louis, Missouri, USA). Antibodies of LC3B (3868), STSQM1/p62 (2324), ULK1(8054T); Atg16L1(8089S) were from Cell Signaling Technology (USA).Antibodies of LAMP1(A2582);Beclin 1(A22361) were from ABclone(China). Antibodies of HA-Tag(1F5C6);Gal3(60207–1-lg);TRIM16 (24403–1-AP);Cathepsin D (21327–1-AP); Cathepsin B (12216–1-AP) were from proteintech (China).Anti-Ubiquitin (ab134953) were obtained from Abcam; Horseradish peroxidase-conjugated secondary antibodies were from Santa Cruz Biotechnology (Santa Cruz, CA, USA), Alexa Fluor 488-conjugated goat anti-mouse secondary antibody (11017), Alexa Fluor 633-conjugated goat anti-mouse secondary antibody (A32728) were obtained from Invitrogen. Chemical reagents LAMP1, MG132 (HY-13259), chloroquine (CQ; HY-17589A), L-Aspartic acid β-hydroxamate (HY-134450), Stretavidin Agarose 6FF (HY-K0218A), were purchased from MedChemExpress; Lysosomal protein extraction kit(Best bio, BB-31452); Cytoplasmic protein extraction kit(Best bio, BB-3113); DLC, CIJ and BTD were synthesized by the research group of Baoxiang Zhao, College of Chemistry, Shandong University.

### Transfection of plasmid and siRNA

The LAMP1 Wild-type plasmid was constructed using standard molecular cloning techniques. Various targeted mutants of LAMP1 were constructed using Quik Change targeted or multisite targeted mutagenesis kits (Stratagene) and confirmed by sequencing. Duplexes of siRNA were synthesized by DynaPro (Qingdao, China).The siRNA with high knockdown efficiency and the optimal concentration were determined by pre-experiment (S10 Fig).The sequence of siRNA targeting against LAMP1 is 5′-GGATATTCACCTTGCGAAA-3′. Cells were spread in 6-well plates 12 hours in advance and DMEM medium containing 2% fetal bovine serum was replaced before transfection. The premixed solution containing plasmid or siRNA was prepared according to the procedure provided by Lipofectamine 3000 (L3000001) and added to the cells at room temperature for 15 minutes, and then incubated in a 37°C carbon dioxide incubator for subsequent experimental operations.

### Virus infection and sample collection

BHK-21 cells grown in 6-well plates were infected with SVA at the multiplicity of infection (MOI) indicated. After 1 h of incubation at 37°C, cells were washed with DMEM medium, then supplemented with DMEM containing 2% FBS. At the indicated times after infection, cells and supernatants were harvested, and the virus solution was collected by lysis of the cells by repeated freeze-thaw. Samples were titrated on BHK-21 cells by using TCID_50_ assay. During the extraction of total cell protein, the cells were scraped off, added with appropriate lysate, cracked on ice for 15 minutes, centrifuged at 12000 × g at 4°C for 10 minutes, and the supernatant was stored at -80°C.

### Transmission electron microscopy experiment

Cells were initially fixed with 2.5% glutaraldehyde in 0.1 M phosphate buffer, pH 7.4, and postfixed with 1% osmium tetroxide. After dehydration through a graded ethanol series, the samples were permeated and embedded in Epon 812 resin. Ultrathin sections (70 nm) were cut using an ultramicrotome, mounted on copper grids, and double stained with uranyl acetate and lead citrate. The prepared sections were subsequently examined under a transmission electron microscope operating at 80 kV.

### Sample enrichment and detection of sulfenylated protein

Cells to be collected were added with a new benzothiadiazine probe (called BTD)1mM incubated at 37°C for 1h. Protein samples were collected and quantified by BCA method. Streptomycin affinity and heterosaccharides magnetic beads were added and incubated at 4°C for 30 min, the protein samples were cleaned, and the supernatant was obtained by centrifugation at 1700 × g, and then 1μL CuAAC mixture was added (Azidobiotin 0.2 mM, TBTA ligand 0.1 mM, CuSO_4_ solution 1.0 mM, sodium ascorbate 1.0 mM). Incubation was performed at room temperature for 2 h, and 4 × Loading Buffer was added. Western blot analysis was performed after metal bath at 100°C.

### Real-time fluorescent qPCR

RNA was harvested using the Simply P Total RNA Extraction Kit (BioFlux China) at 48 hpi. After drug treatment and virus infection, cell RNA was collected. The HiScript III All-in-one RT SuperMix Perfect for qPCR (Vazume, R333) reversely transcribed RNA into cDNA. cDNA is used as a template for qPCR reaction. Real-time fluorescence quantitative PCR by SYBR Green qPCR. The target gene primer sequence is shown in S1 Table.The reaction procedure is as follows, Amplification procedure: pre-denaturation at 95°C for 2 min then denaturation at 95°C for 5s, annealing at 60°C for 20s, extension at 72°C for 10s with 40 cycles; dissolution procedure: 95°C 5s, 60°C 20s, 95°C, continued.

### Western blot analysis

After treatment, cells were lysed in RIPA lysis buffer (P0013, Beyotime, Shanghai, China). Protein content was determined by use of the BCA Protein Assay Kit (P0011, Beyotime, Shanghai, China). Proteins were separated by 12% SDS-PAGE at 4°C and then transferred to the PVDF membrane (IPFL00010, Millipore, Burlington, MA, USA). The membranes were incubated with primary antibodies, then horseradish peroxidase-linked secondary antibodies, and detected using an enhanced chemiluminescence detection kit (Thermo Fisher, 34080). The relative quantity of proteins was analyzed by Image J and normalized to loading controls.

### Immunofluorescence experiment

After treatment, cells were fixed in 4% paraformaldehyde for 15 min. After washing three times with 1 × PBS cells were permeated with 0.1%–0.2% TritonX-100 for 5 min, and then blocked with 5% donkey serum for 30 min. Then cells were incubated with primary antibodies overnight at 4°C and then incubated cells with corresponding secondary antibodies for 60 min at 37°C. Fluorescence intensity was detected by laser scanning confocal microscopy (Zeiss LSM900, Carl Zeiss Canada).

### Lysosomes and Lysosomal pH measurement

Lysosomal staining was performed using LAMP1 primary antibody. LAMP1-positive lysosomes were visualized with confocal microscopy equipment (Zeiss LSM900, Carl Zeiss Canada); Cells were incubated with LysoSensor Green DND-189 (Thermo Fisher Scientific, L7535) for 30 min at 37° C, and their fluorescence was detected by confocal microscopy; the cells were treated with the cell lysosomal membrane permeability (LMP)/ integrity acridine orange fluorescence detection kit (HL10387.1), the lysosomal permeability and permeability changes were observed under a laser confocal microscope at 555 nm excitation wavelength, and the photos were analyzed.

### β-Hexosaminidase (β-HEX) activity assay

The cell supernatant and lysosomal fraction were extracted, and the enzyme activity was detected according to the procedure shown in the mouse β-hexosaminidase (β-Hex) ELISA kit. The absorbance value of the sample was measured at 450 nm using a microplate reader, and the enzyme activity (U/mL) was determined according to the standard curve.

### Molecular docking

UniProt Knowledgebase (https://www.uniprot.org/), as a hub of integrated protein data, was selected to download 3D structures of molecules. The UniProt identifiers of LAMP1 protein were downloaded (P11279). Protein docking of LAMP1 and DLC molecules was performed. The input parameters are selected by default and possible surface residues around active residues within a radius of 6.5A are screened. Docking parameters, including distance constraints, clustering parameters, dihedral and hydrogen bond constraints, symmetry constraints, and energy and interaction parameters, are also set to default values. Use PyMOL software (http://www.pymol.org) to visualize molecular graphics.The implementation code (https://pymolwiki.org/index.php/InterfaceResidues) and through InterfaceResidues automatic docking of protein molecules.

### Microscale Thermophoresis(MST)

The target protein LAMP1 and the ligand DLC(20 μM) to be tested were prepared and labeled with fluorescent dye. A series of experiments with ligand concentration gradients were performed by adding the mixed samples to glass capillaries in the MST experimental equipment (each capillary loaded with 20μL of sample) and placing the sample loaded capillary into the MST instrument (NanoTemper,Monolith NT.115). The hot vortex data were collected by heating a part of the capillary and measuring the migration of molecules. The MST software was used to analyze the experimental data, draw the molecular binding curve, and calculate the binding affinity (KD) value.

### In vivo virus infection and drug treatment experiments in BALB/c mice

Healthy BALB/c mice aged 4–6 weeks were randomly divided into 4 groups with 5 mice in each group. Each group was treated according to the following protocol: control group: normal saline was given. Challenge group: Oral inoculation of SVA-CH-SDGT-2017 with 1 × 10^-7^ TCID_50_ was performed only for viral infection without drug treatment. Low-dose administration group: SVA-CH-SDGT-2017 of 1 × 10^-7^ TCID_50_ was orally inoculated with low-dose DLC (2 mg/kg) of antiviral drug for 7 days. High-dose administration group: SVA-CH-SDGT-2017 of 1 × 10^-7^TCID_50_ was orally inoculated with high-dose DLC (5 mg/kg) of antiviral drug for 7 days. After challenge, all animals were monitored daily for clinical signs. At 21 dpi, all mice in each group were euthanized and the heart, lungs, liver, spleen, kidneys, and serum were collected from each mouse. Each organ was cut into two parts, one for histopathological analysis and the other for quantifying the viral load of SVA by SYBR GREEN quantitative real-time polymerase chain reaction (qRT-PCR). All animals used in this study were made to minimize suffering and euthanasia.

### LC-MS/MS

After cell culture, the cell lysate was obtained, the samples were first reduced alkylated and treated with Trypsin. After that, the treated samples were analyzed by LC-MS/MS. The conditions for liquid chromatography were as follows: 75μm i.d. × 250 cm, NanoViper C181.9 μm; Mobile phase A: 0.1% formic acid; Mobile phase B: 0.1% formic acid, 80% ACN; Flow rate: 300nL/min; Analysis time of each component:66min.Primary mass spectrum parameters: Resolution:120,000AGC target: Standard; Maximum IT: custom; Scanrange: 300 to 1800m/z secondary mass spectrumparameters:Resolution:15000;AGCtarget:Standard;MaximumIT:customNCE/stepped NCE:30.The raw file of the original mass spectrometry results is obtained, and the software Byonic is used to analyze and match the data to obtain the identification result.

### Statistical analysis

Results are reported as mean ± SEM. All experiments were repeated at least three times independently. For statistical analysis, Graph Pad Prism software (version 8.0) was used. Statistical comparisons for data were performed using one-way analysis of variance (ANOVA) followed by Tukey: compare all pairs of columns test. “*” means p < 0.05, “**”means p < 0.01 and “***” means p < 0.001.

### Reproducibility

Detailed step-by-step protocols for key experiments, including cell infection, immunofluorescence, immunoelectron microscopy, and animal studies, are available at protocols.io (DOI: https://dx.doi.org/10.17504/protocols.io.14egnrk9zl5d/v1).

## Supporting information

S1 Fig Chemical structure diagram of DLC. A deep-red probe (named DLC) for effective bioimaging of bisulfite was developed from flavone moiety and benzoindole derivative based on intramolecular charge transfer (ICT) and Förster resonance energy transfer (FRET) platform.
(TIF)

S2 Fig SVA infection affected the protein expression of LAMP2 and LAMP3. (A)After SVA infection, BHK-21 cells were collected at different time points to detect the expression levels of LAMP2 and LAMP3.(B, C)The results of Western blot were analyzed by gray scale.(ns, p > 0.05, *p < 0.05, ***p < 0.001, n = 3).
(TIF)

S3 Fig β-hexosaminidase (β-HEX) activity of cell supernatant and lysosomal fractions after SVA infection was determined. (A) The OD value of the measured standard was used as the abscissa and the concentration of the standard The degree value is the ordinate, the standard curve is drawn, and the linear regression equation is obtained.(B-C)The supernatant and lysosomal fractions of cells infected with SVA at different time points were collected, and the enzyme activity (U/mL) was measured according to the standard curve according to the procedure shown in the kit. The data were collected in triplicate for statistical analysis.(ns, p > 0.05, *p < 0.05, ***p < 0.001, n = 3).
(TIF)

S4 Fig Effect of DLC on cell viability. (A, B)Different concentrations of DLC (0, 0.5, 1, 2.5, 5, 10 μM)were added to PK-15 and BHK-21 cells, and after 24 h, the light absorption value at 540 nm was measured by CCK-8 method to calculate cell viability. (ns, p > 0.05, n = 3).
(TIF)

S5 Fig Sulfenylated protein in whole cell lysate enriched by BTD probe method. Cells were incubated with DMSO and BTD probe (1 mM) separately for 1 h, either infected or uninfected with SVA(0.01 MOI), and with or without the use of DLC (5 μM). The total sulfenylation levels in the cells were then detected using a click chemistry-based biotin crosslinking method to validate the feasibility of the BTD probe approach.(*p < 0.05, **p < 0.01, ***p < 0.001, n = 3).
(TIF)

S6 Fig Cys375 is one of the non-lysine ubiquitination sites of LAMP1. (A-D)The nine cysteines of LAMP1, which were mutated to alanine, were infected with SVA and treated with DLC, respectively, and cellular proteins were collected to examine the level of ubiquitination of LAMP1 protein in each sample. DLC significantly reduced the ubiquitination of WTLAMP1, but not C375A-LAMP1. Moreover, the overall ubiquitination of C375ALAMP1 was down-regulated compared with WT-LAMP1. (E-G) The ubiquitin modification of LAMP1 Cys375 after SVA infection was identified by LC-MS/MS in BHK-21, PK-15, HEK-293t cells. In the secondary mass spectrometry, C/K [+114.043]: ubiquitination modification occurred on cysteine, and the molecular weight changed to +114.043Da.(*p < 0.05, **p < 0.01, ***p < 0.001, n = 3).
(TIF)

S7 Fig Total ion current chromatogram of the sample. (A-C)Total ion current chromatogram of LAMP1 Cys ubiquitination modification in BHK-21, PK-15, and HEK-293t cells after SVA infection as determined by LC-MS/MS.
(TIF)

S8 Fig LAMP1 cysteine ubiquitination site identified by LC-MS/MS. Secondary mass spectrogram of ubiquitination on LAMP1 cysteine detected by LC-MS/MS. (TIF)

S9 Fig DLC reduced the cytopathic effect (CPE) induced by SVA. The virus solution was added according to MOI0.01 and infected at 37°C for 1h. The virus solution was discarded and replaced with DMEM containing different concentrations of DLC. The cells were cultured at 37°C and 5%CO_2_ for 24 h to observe the pathological changes. Scale bar: 50 μM.
(TIF)

S10 Fig DLC reduced the viral load of SVA in cells(A)The SVA plasmid was used as standard for gradient dilution and template for RT-qPCR reaction. The standard curve was plotted with the logarithm of gene copy number as abscissa and the Ct value as ordinate: Ct = -3.01lgX-44.77. B: 36 h after virus infection, cells treated with DLC(0 μM, 2 μM, 5 μM, 10 μM) were collected, RNA was extracted and detected by RT-qPCR, and virus copy number was calculated.(ns, p > 0.05, *p < 0.05, ***p < 0.001, n = 3).
(TIF)

S11 Fig The interference effect after transfection was detected. Different concentrations (10, 30, and 50nM) of control (Scramble) and LAMP1 siRNA were added according to the method provided by the liposome transfection kit, respectively. After 48 hours of continuous culture, cellular protein was collected to detect the protein level of LAMP1, and the results were statistically analyzed.(ns,p > 0.05, ***p < 0.001, n = 3).
(TIF)

S12 Fig DLC treatment reduced VP2 content in various organ tissues of mice. (A,B)VP2 immunohistochemical sections and statistical analysis of heart tissues of mice in SVA infection and DLC (2mg/kg) injection groups; (C, D)VP2 immunohistochemical sections and statistical analysis of the liver of mice in the SVA infection and DLC (2mg/kg) injection groups;(E,F)VP2 immunohistochemical sections and statistical analysis of spleen of mice in SVA infection and DLC (2mg/kg) injection groups;(G, H) VP2 immunohistochemical sections and statistical analysis of the lungs of mice in the SVA infection and DLC (2mg/kg) injection groups; (I, J) VP2 immunohistochemical sections and statistical analysis of kidneys from mice infected with SVA and injected with DLC (2mg/kg), Scale bar: 100 μm. (*p < 0.05, **p < 0.01, n = 5).
(TIF)

S1 Table Primer sequences of target genes. (DOCX)

S2 Table Mass spectrometry coverage analysis of WT-LAMP1 and C375ALAMP1 ubiquitination. (DOCX)

S1 Raw Images Original picture of the western blots.pdf (PDF)

S1 Data MST-DLC-LAMP DoseResponseRawDataGrouped. Fig 1(A)VP2,LAMP1,p62,LC3,ACTB.(F)pro-CTSD,mature-CTDSD,pro-CTDB,mature-CTSB,ACTB; Fig 2(A)LAMP1,ACTB(C)LAMP1,ACTB(DLAMP1,ACTB)(L)LAMP1,VP2,LC3,ACTB; Fig 3(A)VP2,LC3,ACTB(F)ULK,ATG16L1,Beclin1,ACTB. Fig 4 (A)Ub,LAMP1,ACTB(B)LAMP1,ACTB(E)LAMP1,ACTB(G)Gal3,TRIM16,input,ACTB. Fig 6(D)Cys-SOH-LAMP1,ACTB(F)Cys-SOH-LAMP1,ACTB(H)Cys-SOH-LAMP1,ACTB(G)VP2,ACTB. Fig 7.(A)VP2,ACTB(G)LAMP1,VP2,ACTB. S2 Fig.LAMP2,LAMP3,ACTB. S5 Fig.Cys-SOH,GAPDH; S6 Fig.Ub;HA,LAMP1,ACTB. S11 Fig.siLAMP1,ACTB.
(XLSX)

S2 Data Statistical data.docx (Table.1-Table.50). (DOCX)
