# Supplementary figures and images for: A novel SO2 probe inhibits lysophagy induced by Senecavirus A infection by promoting LAMP1 Cys375 sulfenylation
Source: PLoS Pathog. 2026 Feb 5;22(2):e1013932. doi: 10.1371/journal.ppat.1013932 (PMC12875573; doi:10.1371/journal.ppat.1013932)

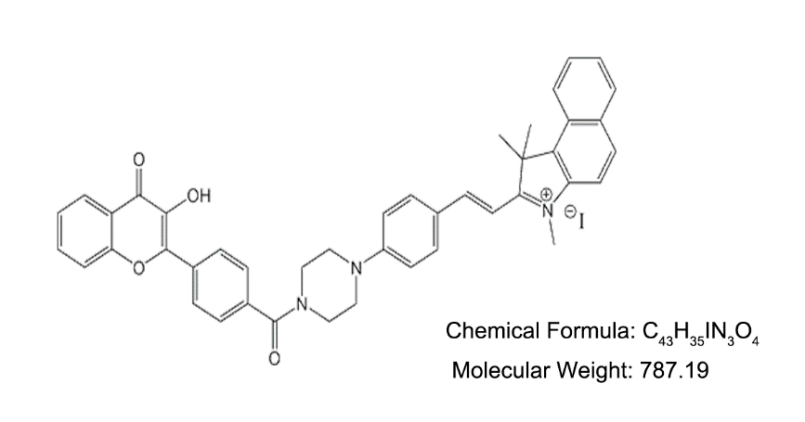

Supplement: S1 Fig — A deep-red probe (named DLC) for effective bioimaging of bisulfite was developed from flavone moiety and benzoindole derivative based on intramolecular charge transfer (ICT) and Förster resonance energy transfer (FRET) platform. (TIF) [file ppat.1013932.s001.tif]

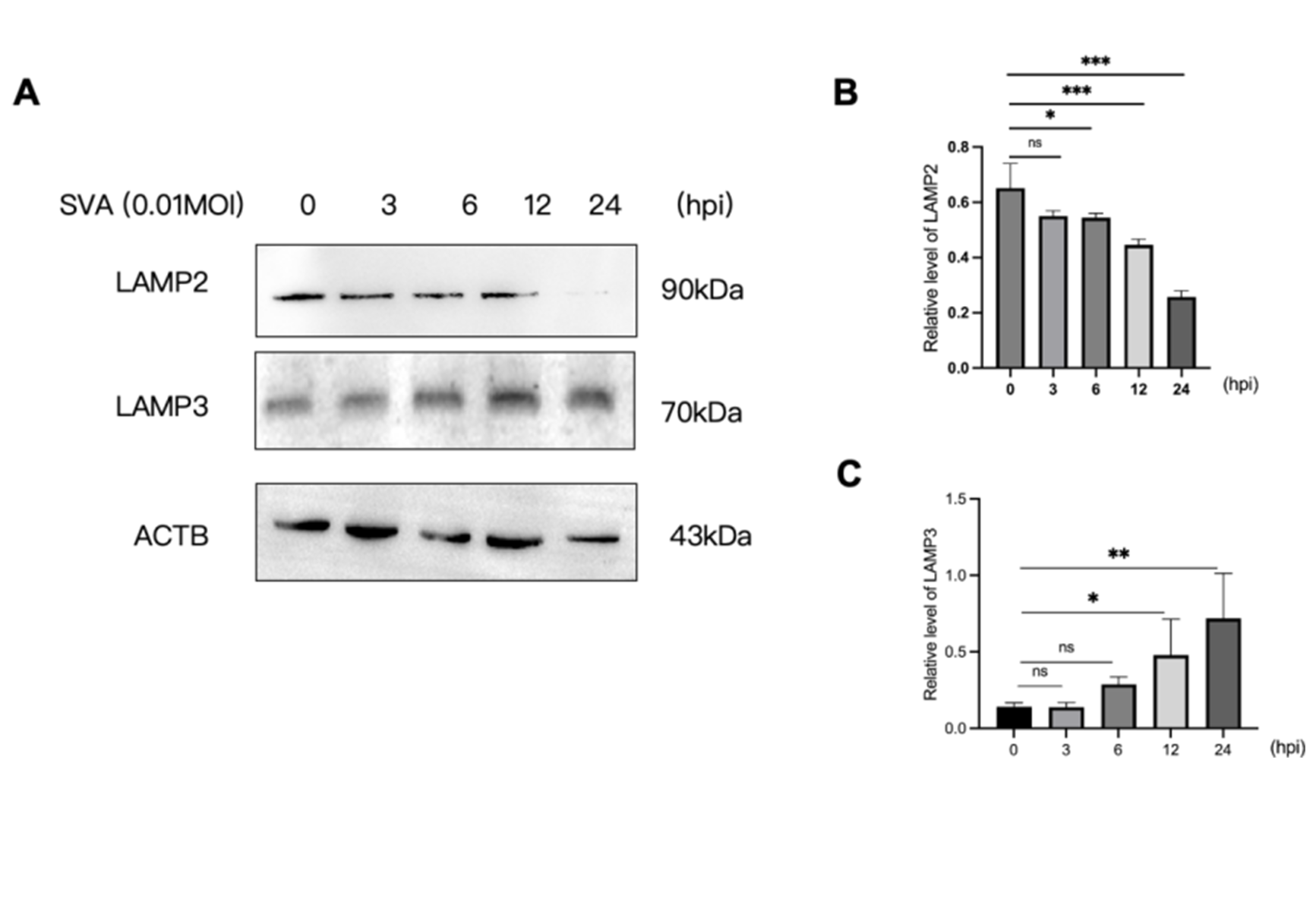

Supplement: S2 Fig — (A)After SVA infection, BHK-21 cells were collected at different time points to detect the expression levels of LAMP2 and LAMP3.(B, C)The results of Western blot were analyzed by gray scale.(ns, p > 0.05, *p < 0.05, ***p < 0.001, n = 3). (TIF) [file ppat.1013932.s002.tif]

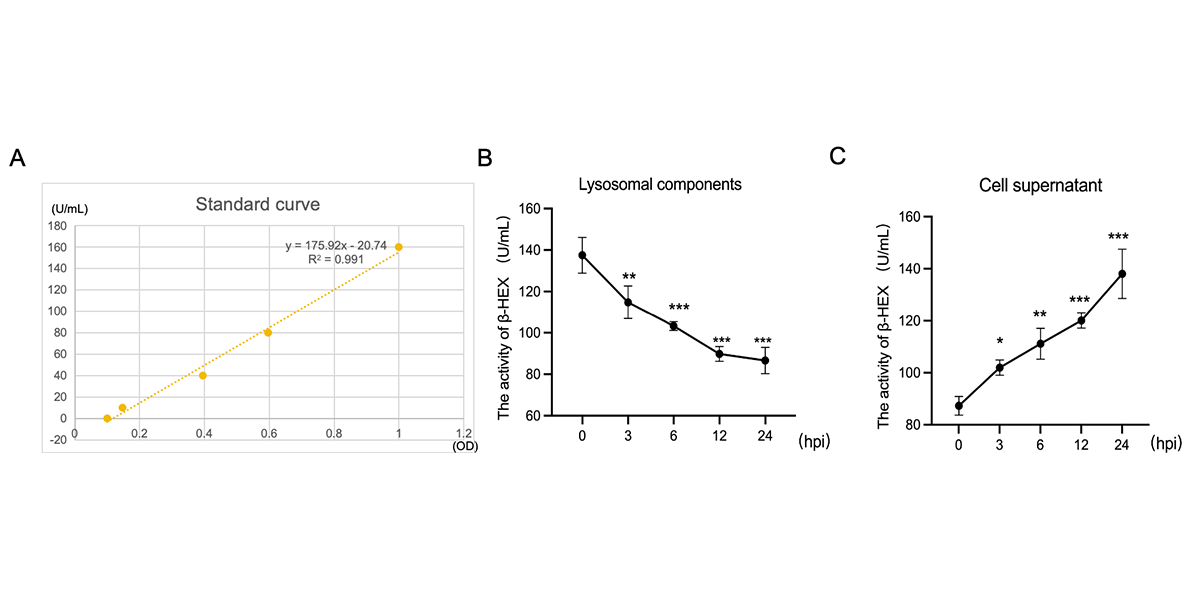

Supplement: S3 Fig — β-hexosaminidase (β-HEX) activity of cell supernatant and lysosomal fractions after SVA infection was determined. (A) The OD value of the measured standard was used as the abscissa and the concentration of the standard The degree value is the ordinate, the standard curve is drawn, and the linear regression equation is obtained.(B-C)The supernatant and lysosomal fractions of cells infected with SVA at different time points were collected, and the enzyme activity (U/mL) was measured according to the standard curve according to the procedure shown in the kit. The data were collected in triplicate for statistical analysis.(ns, p > 0.05, *p < 0.05, ***p < 0.001, n = 3). (TIF) [file ppat.1013932.s003.tif]

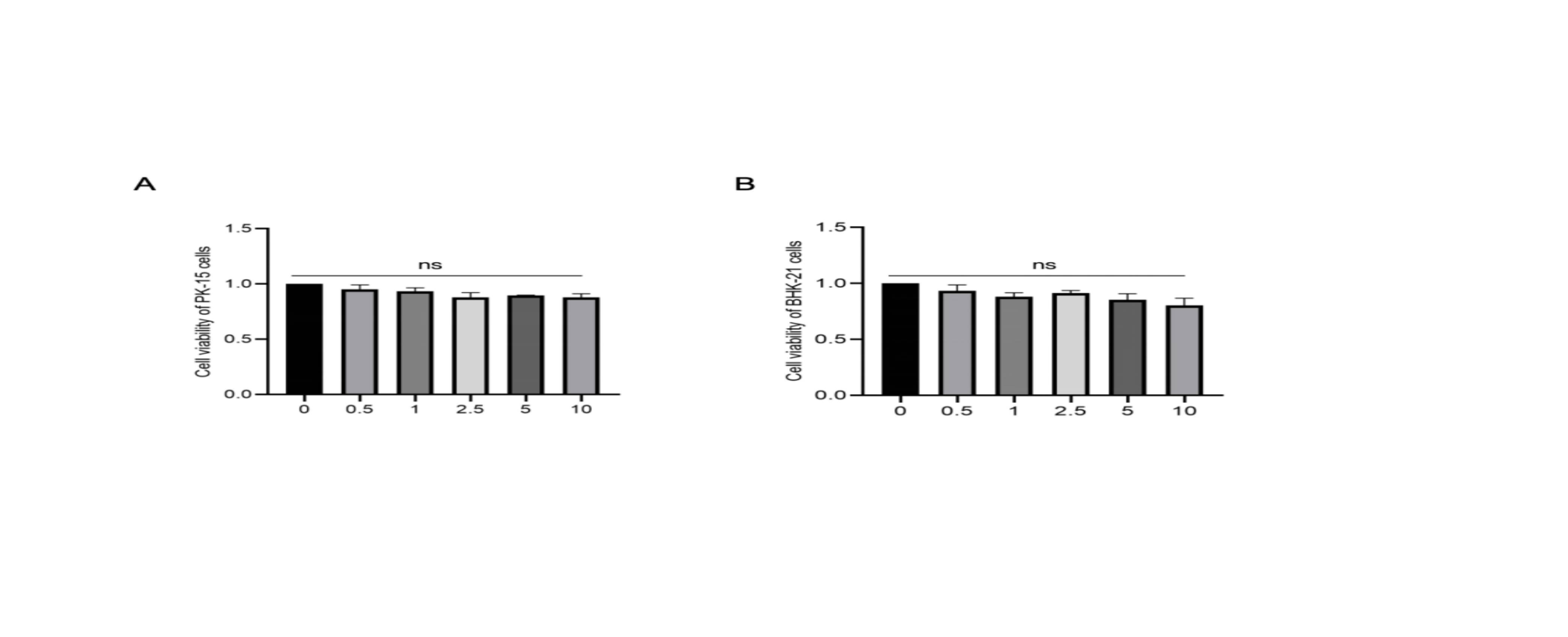

Supplement: S4 Fig — (A, B)Different concentrations of DLC (0, 0.5, 1, 2.5, 5, 10 μM)were added to PK-15 and BHK-21 cells, and after 24 h, the light absorption value at 540 nm was measured by CCK-8 method to calculate cell viability. (ns, p > 0.05, n = 3). (TIF) [file ppat.1013932.s004.tif]

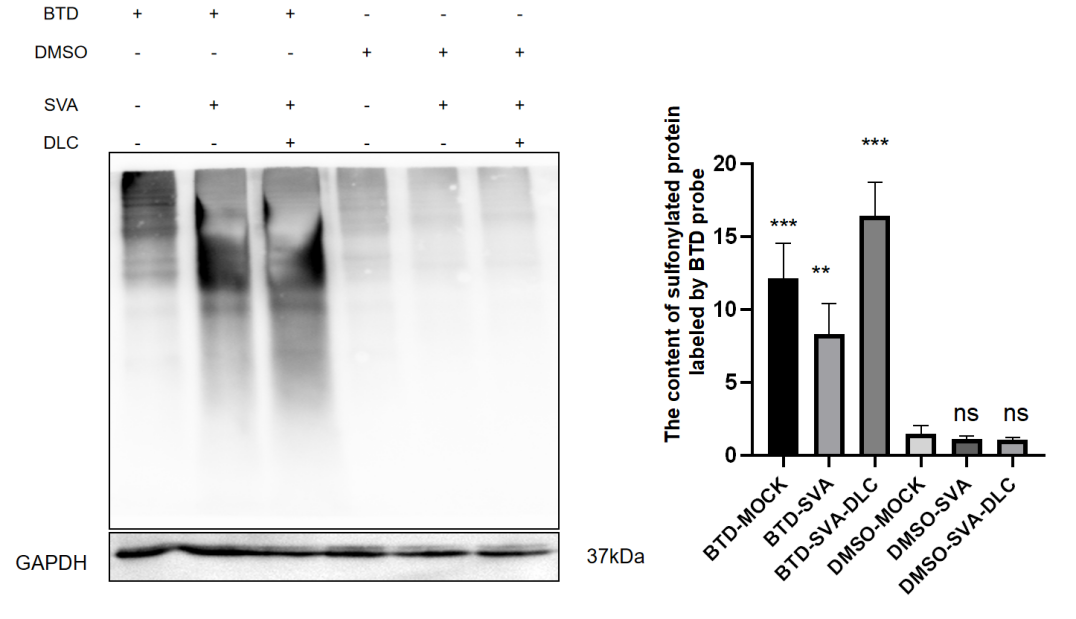

Supplement: S5 Fig — Cells were incubated with DMSO and BTD probe (1 mM) separately for 1 h, either infected or uninfected with SVA(0.01 MOI), and with or without the use of DLC (5 μM). The total sulfenylation levels in the cells were then detected using a click chemistry-based biotin crosslinking method to validate the feasibility of the BTD probe approach.(*p < 0.05, **p < 0.01, ***p < 0.001, n = 3). (TIF) [file ppat.1013932.s005.tif]

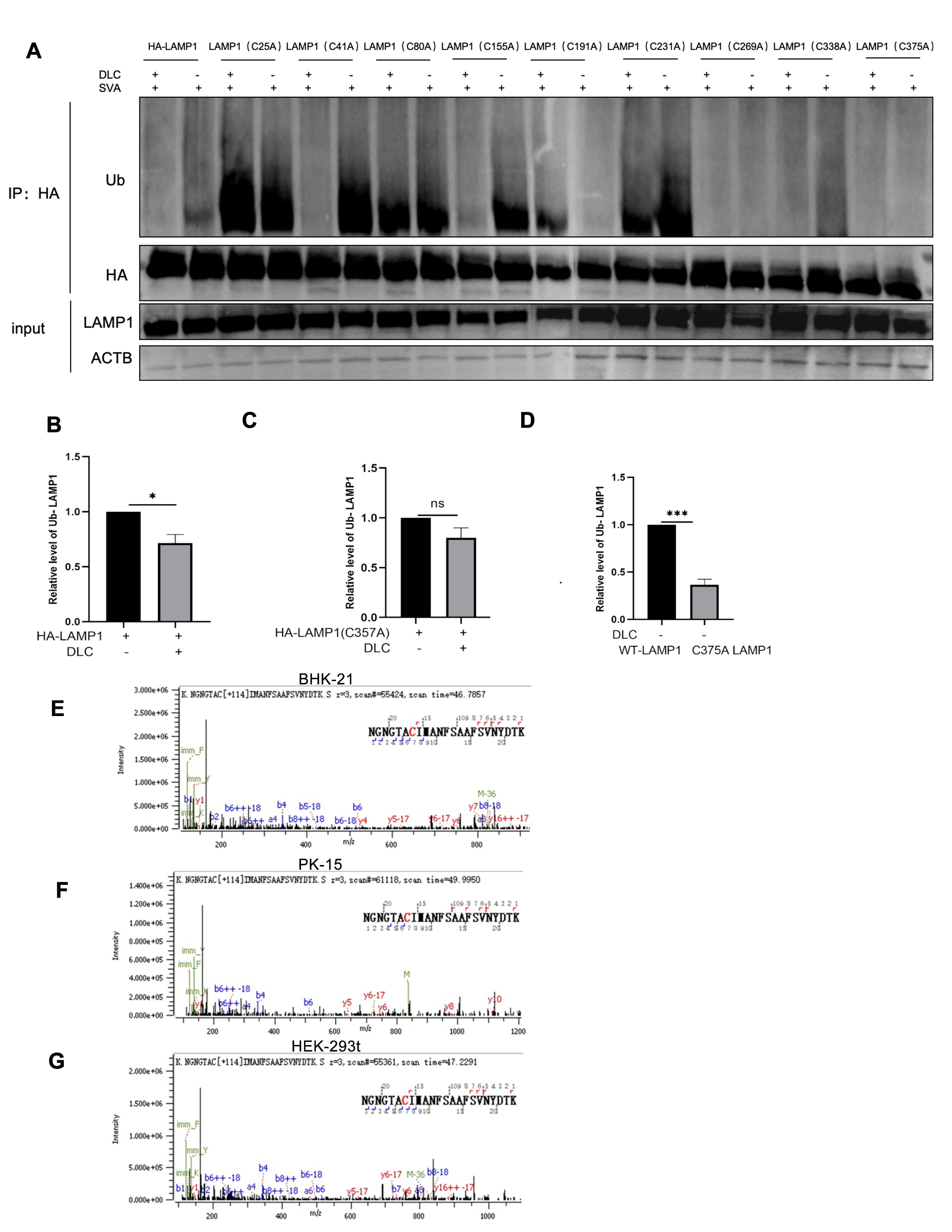

Supplement: S6 Fig — (A-D)The nine cysteines of LAMP1, which were mutated to alanine, were infected with SVA and treated with DLC, respectively, and cellular proteins were collected to examine the level of ubiquitination of LAMP1 protein in each sample. DLC significantly reduced the ubiquitination of WTLAMP1, but not C375A-LAMP1. Moreover, the overall ubiquitination of C375ALAMP1 was down-regulated compared with WT-LAMP1. (E-G) The ubiquitin modification of LAMP1 Cys375 after SVA infection was identified by LC-MS/MS in BHK-21, PK-15, HEK-293t cells. In the secondary mass spectrometry, C/K [+114.043]: ubiquitination modification occurred on cysteine, and the molecular weight changed to +114.043Da.(*p < 0.05, **p < 0.01, ***p < 0.001, n = 3). (TIF) [file ppat.1013932.s006.tif]

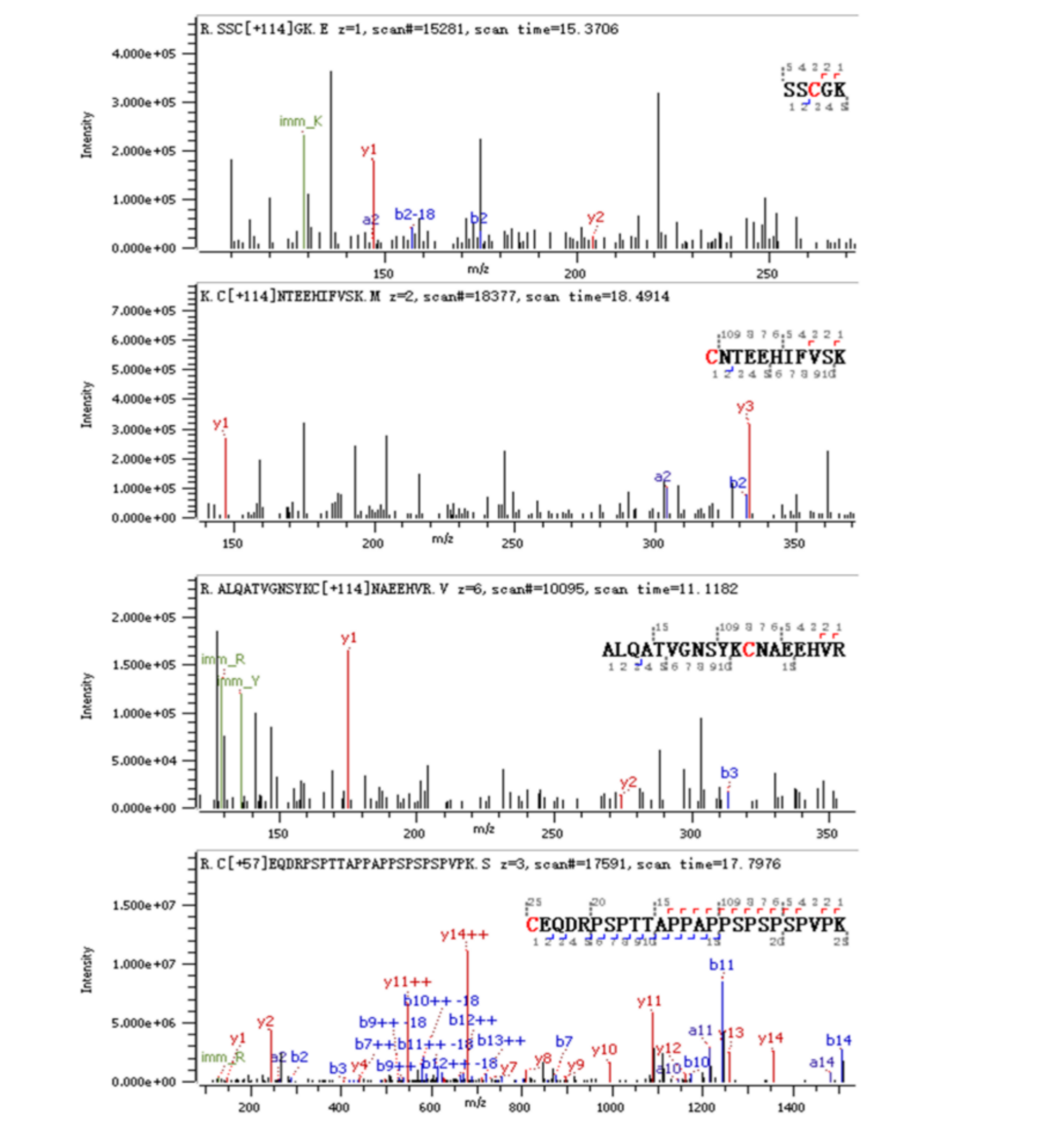

Supplement: S8 Fig — (TIF) [file ppat.1013932.s008.tif]

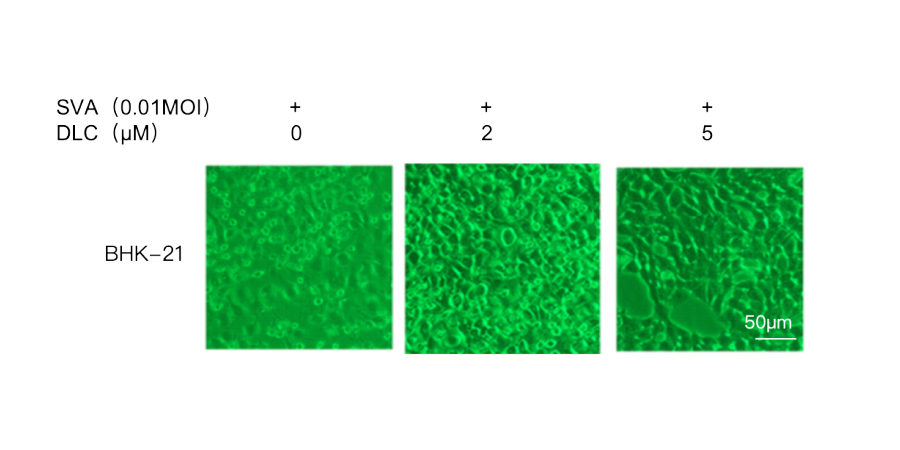

Supplement: S9 Fig — The virus solution was added according to MOI0.01 and infected at 37°C for 1h. The virus solution was discarded and replaced with DMEM containing different concentrations of DLC. The cells were cultured at 37°C and 5%CO2 for 24 h to observe the pathological changes. Scale bar: 50 μM. (TIF) [file ppat.1013932.s009.tif]

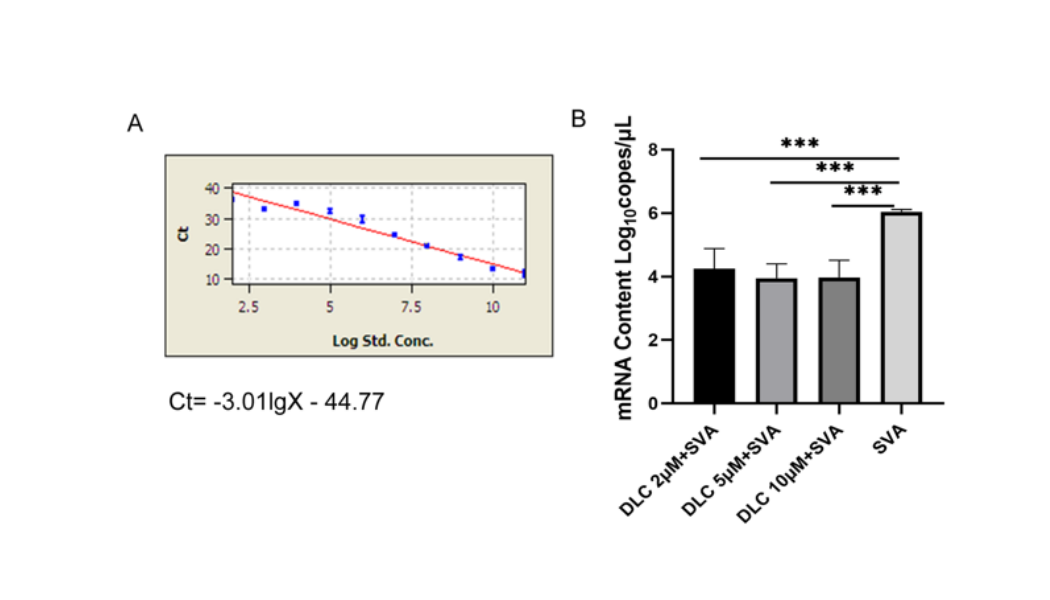

Supplement: S10 Fig — The standard curve was plotted with the logarithm of gene copy number as abscissa and the Ct value as ordinate: Ct = -3.01lgX-44.77. B: 36 h after virus infection, cells treated with DLC(0 μM, 2 μM, 5 μM, 10 μM) were collected, RNA was extracted and detected by RT-qPCR, and virus copy number was calculated.(ns, p > 0.05, *p < 0.05, ***p < 0.001, n = 3). (TIF) [file ppat.1013932.s010.tif]

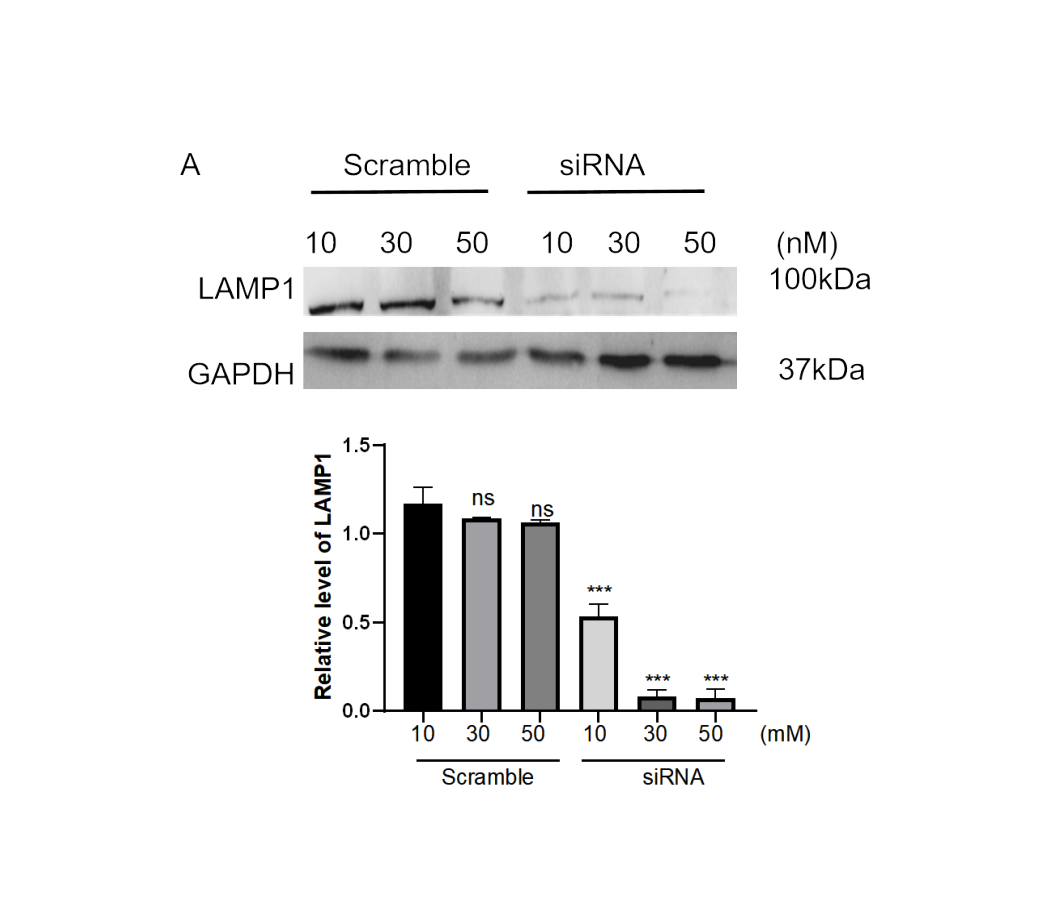

Supplement: S11 Fig — Different concentrations (10, 30, and 50nM) of control (Scramble) and LAMP1 siRNA were added according to the method provided by the liposome transfection kit, respectively. After 48 hours of continuous culture, cellular protein was collected to detect the protein level of LAMP1, and the results were statistically analyzed.(ns,p > 0.05, ***p < 0.001, n = 3). (TIF) [file ppat.1013932.s011.tif]

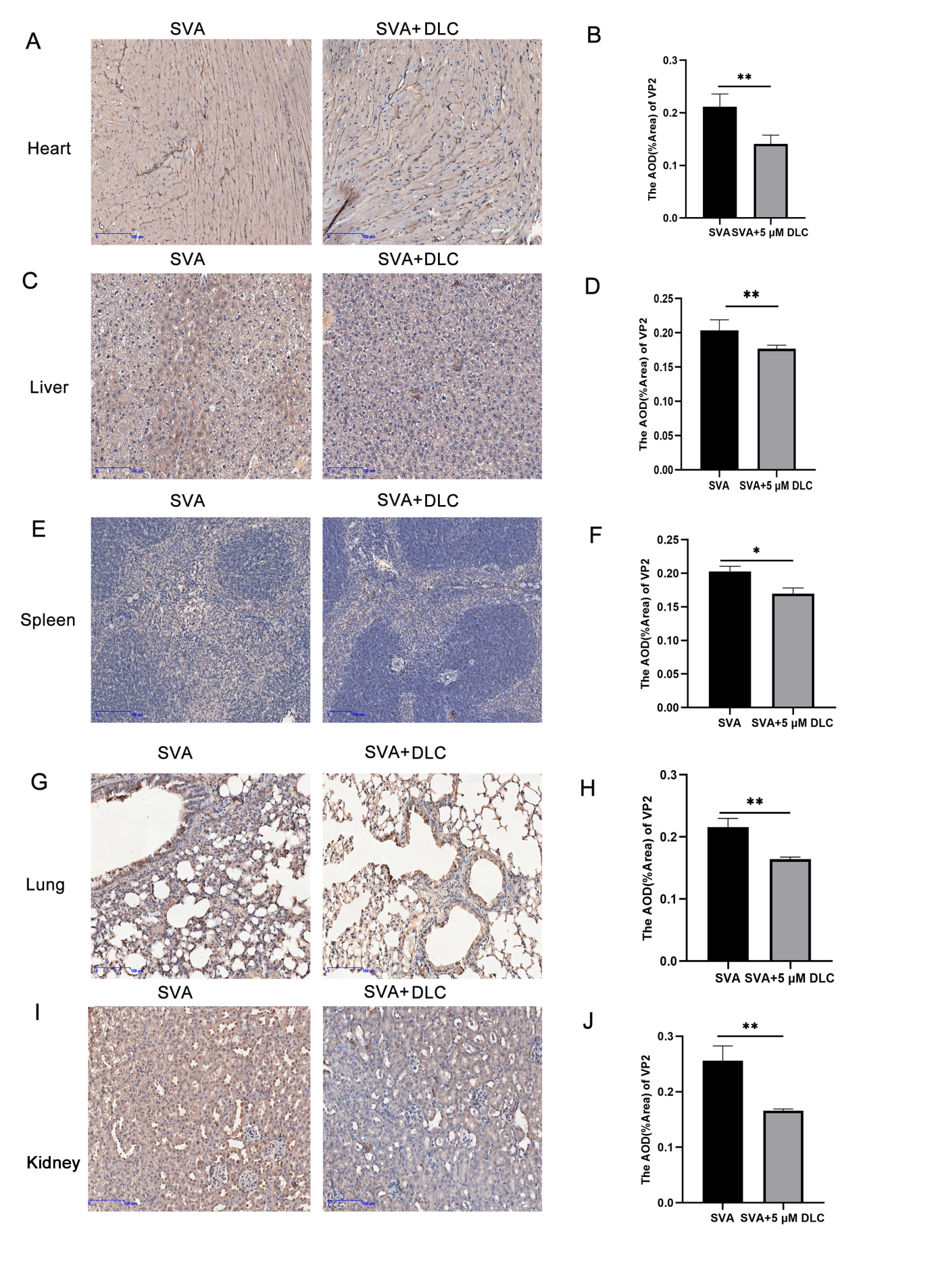

Supplement: S12 Fig — (A,B)VP2 immunohistochemical sections and statistical analysis of heart tissues of mice in SVA infection and DLC (2mg/kg) injection groups; (C, D)VP2 immunohistochemical sections and statistical analysis of the liver of mice in the SVA infection and DLC (2mg/kg) injection groups;(E,F)VP2 immunohistochemical sections and statistical analysis of spleen of mice in SVA infection and DLC (2mg/kg) injection groups;(G, H) VP2 immunohistochemical sections and statistical analysis of the lungs of mice in the SVA infection and DLC (2mg/kg) injection groups; (I, J) VP2 immunohistochemical sections and statistical analysis of kidneys from mice infected with SVA and injected with DLC (2mg/kg), Scale bar: 100 μm. (*p < 0.05, **p < 0.01, n = 5). (TIF) [file ppat.1013932.s012.tif]
