# Supplementary material for: A novel SO2 probe inhibits lysophagy induced by Senecavirus A infection by promoting LAMP1 Cys375 sulfenylation
Source: PLoS Pathog. 2026 Feb 5;22(2):e1013932. doi: 10.1371/journal.ppat.1013932 (PMC12875573; doi:10.1371/journal.ppat.1013932)
Supplement: S1 Table — (DOCX) [file ppat.1013932.s013.docx]

**Table S1. Primer sequences of target genes**

| **Genes** | **Forword (5’→3’)** | **Reverse (5’→3’)** |
| --- | --- | --- |
| LAMP1 | 5’-GACGGTGGAGAGCAAGAACAGTG-3’ | 5’-TCTGGCACCACACCTTCT-3’ |
| SVA VP2 | 5’-AGAATTTGGAAGCCATGCTCT-3’ | 5’-GAGCCAACATAGAAACAGATTGC-3’ |
| β-actin | 5’-TCTGGCACCACACCTTCT-3’ | 5’-ATCTGGGTCATCTTCTCCA-3’ |
