# Supplementary material for: A novel SO2 probe inhibits lysophagy induced by Senecavirus A infection by promoting LAMP1 Cys375 sulfenylation
Source: PLoS Pathog. 2026 Feb 5;22(2):e1013932. doi: 10.1371/journal.ppat.1013932 (PMC12875573; doi:10.1371/journal.ppat.1013932)
Supplement: S2 Table — (DOCX) [file ppat.1013932.s014.docx]

**Table S2. Mass spectrometry coverage analysis of WT-LAMP1 and C375ALAMP1 ubiquitination**

| Name | Coverage% | Avg. Mass | PTM |
| --- | --- | --- | --- |
| WT-LAMP1 | 25 | 44882 | Deamidation (NQ); GlyGly_C；Carbamidomethylation |
| C375ALAMP1 | 10 | 44882 | Deamidation (NQ); |

WT-LAMP1 TIC diagram

C375A-LAMP1 TIC diagram
