# Supplementary material for: A novel SO2 probe inhibits lysophagy induced by Senecavirus A infection by promoting LAMP1 Cys375 sulfenylation
Source: PLoS Pathog. 2026 Feb 5;22(2):e1013932. doi: 10.1371/journal.ppat.1013932 (PMC12875573; doi:10.1371/journal.ppat.1013932)

Fig1

A

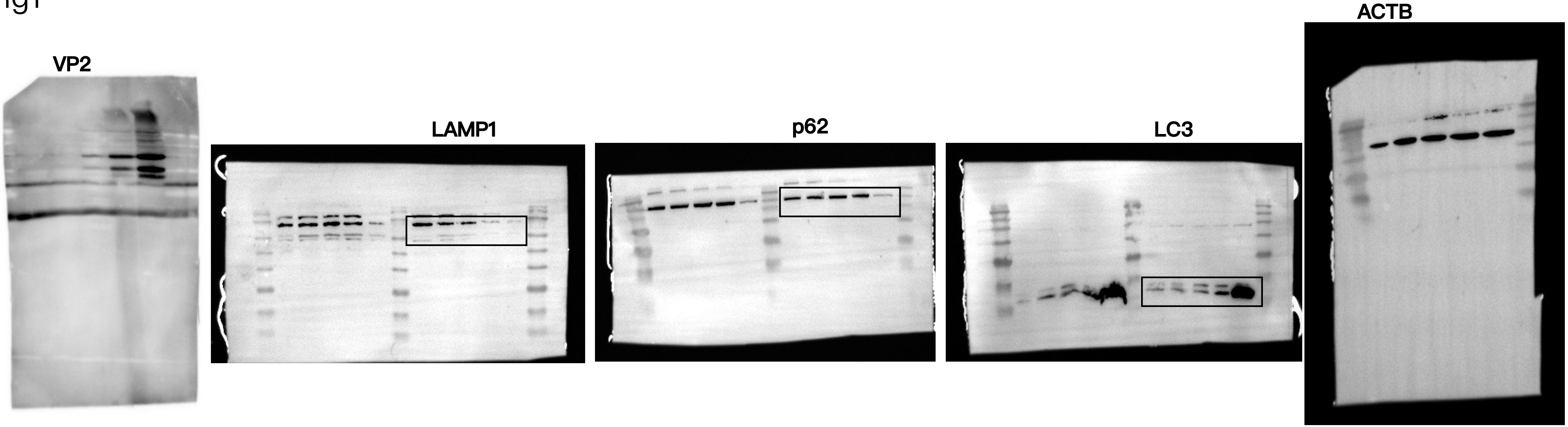
VP2
LAMP1
p62
LC3
ACTB

F

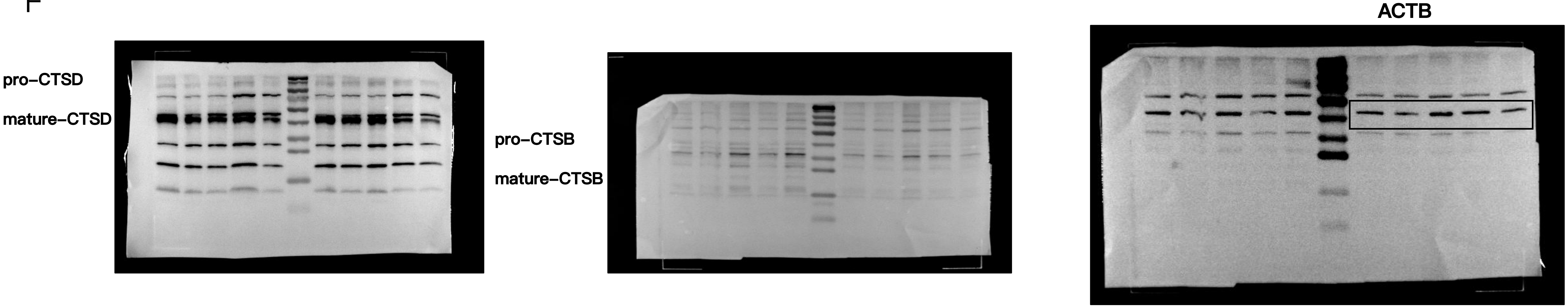
pro-CTSD
mature-CTSD
pro-CTSB
mature-CTSB
ACTB

Fig2

A

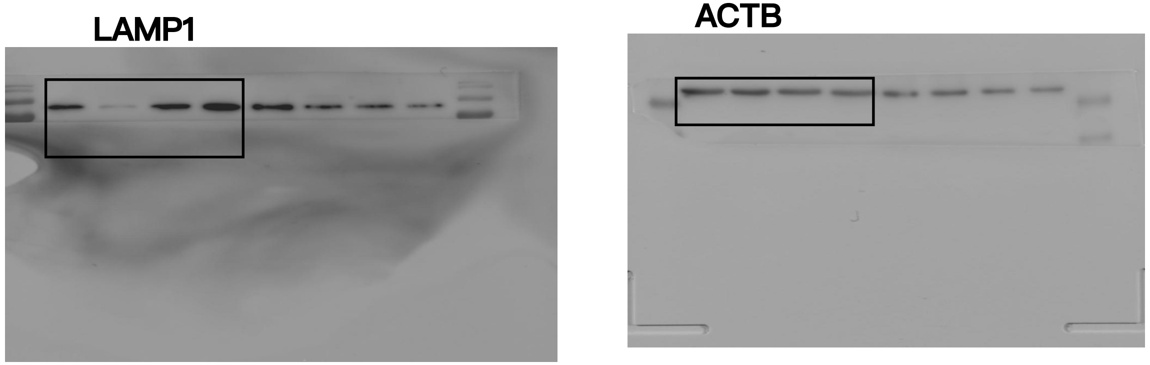

C

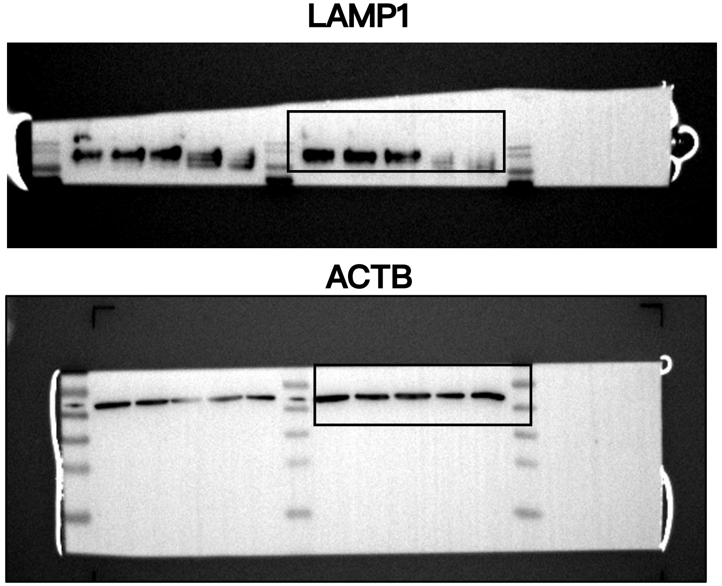

D

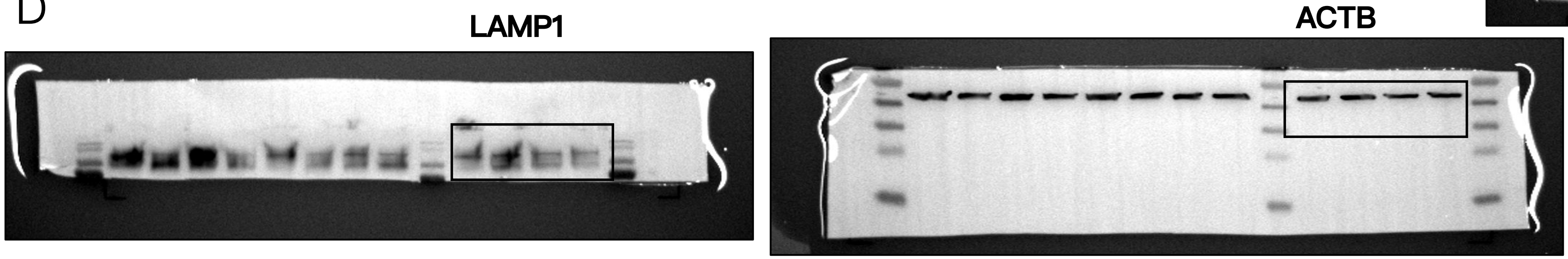

L

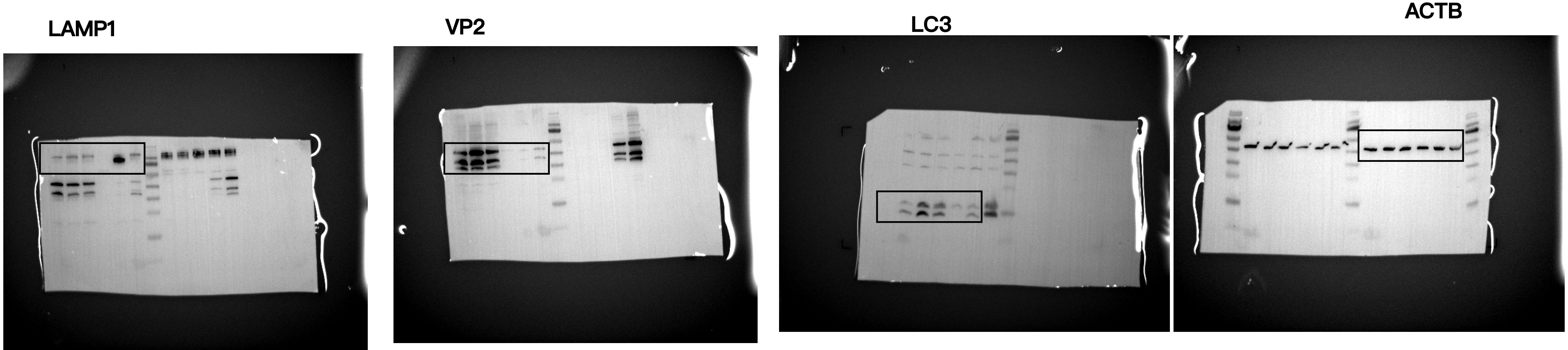

Fig3

A

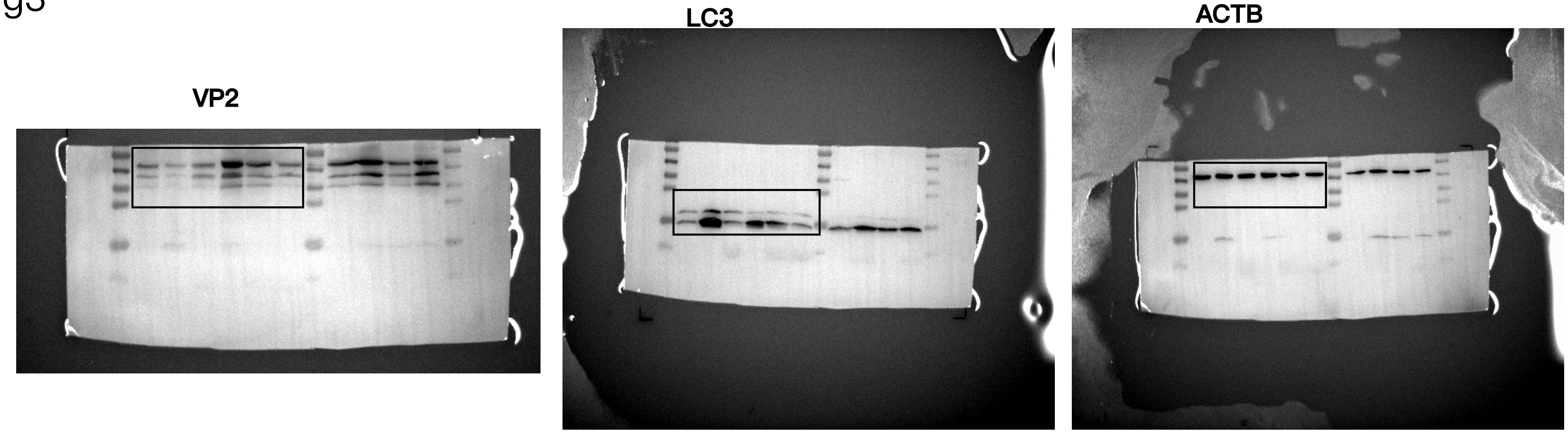

F

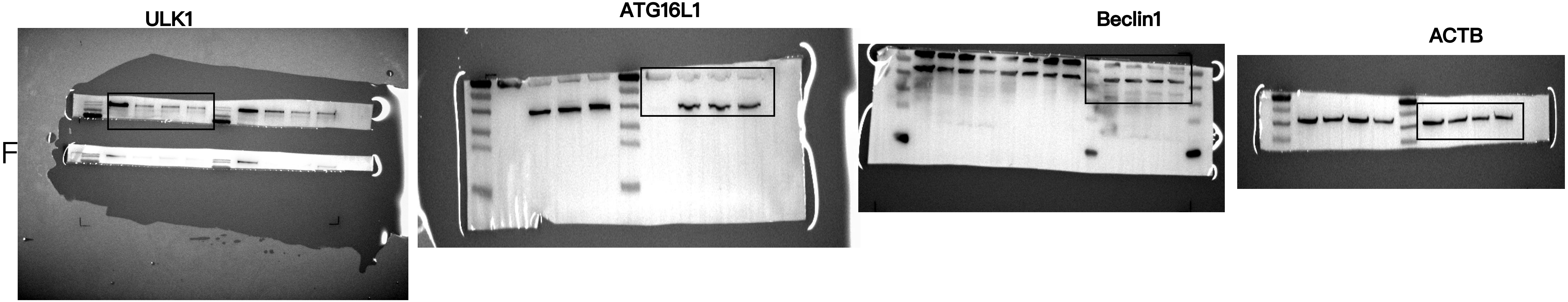

Fig 4

A

Ub

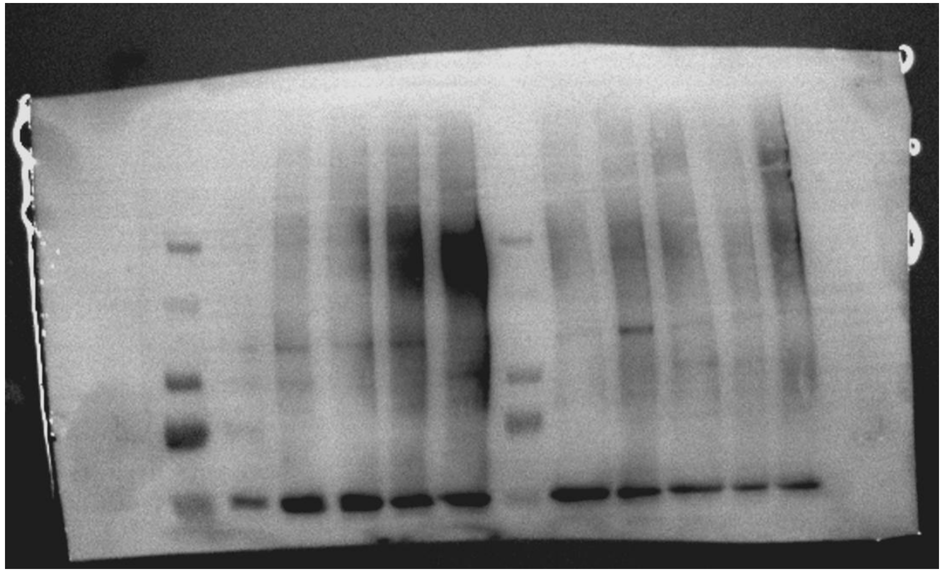

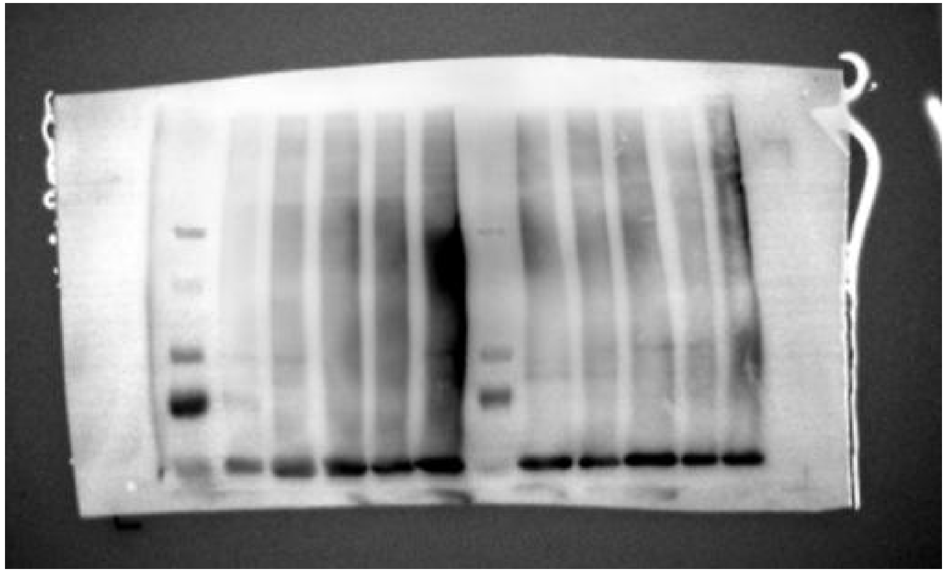

LAMP1

ACTB

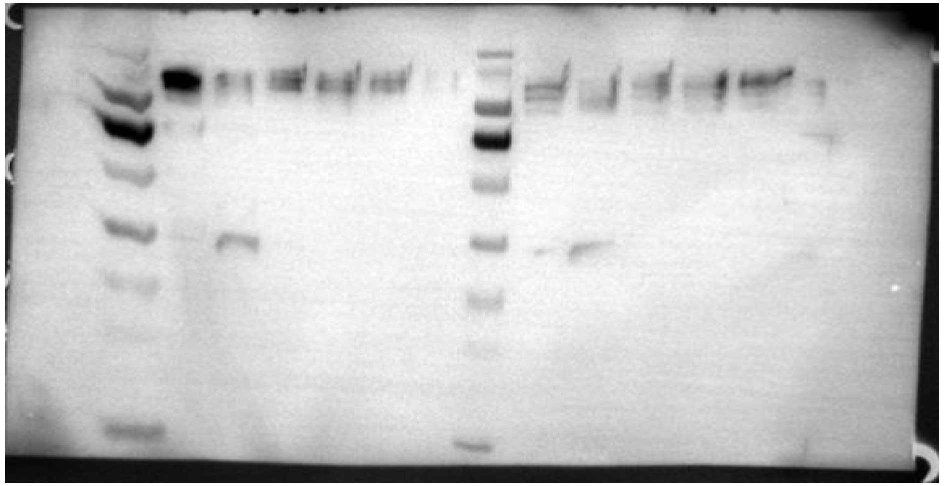

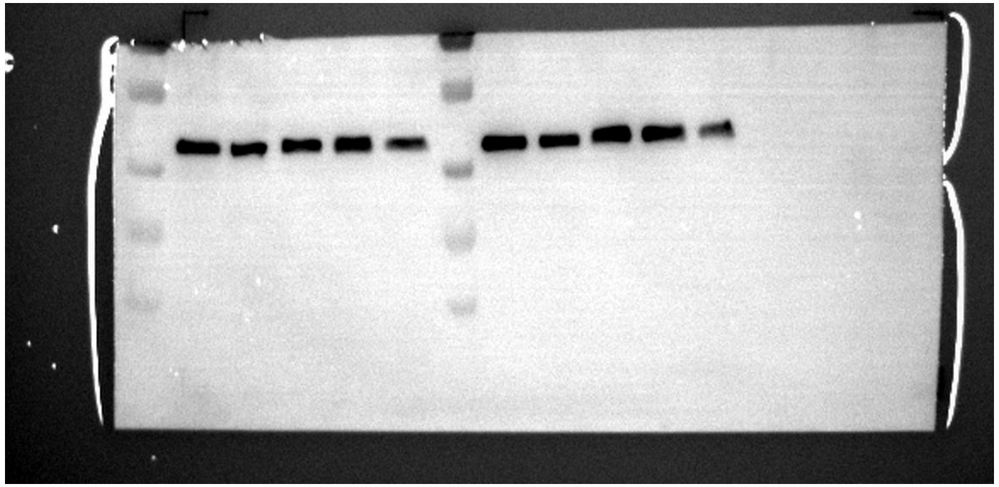

Fig 4

B

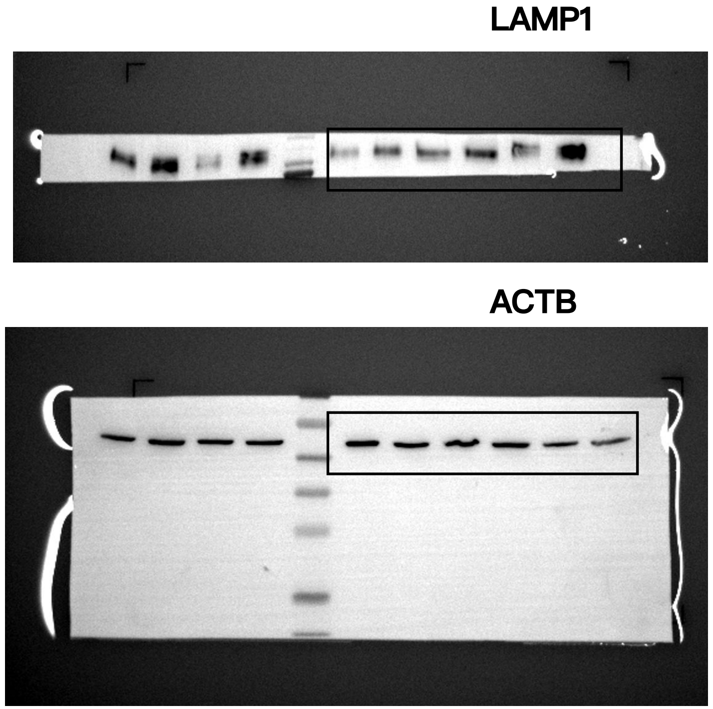

E

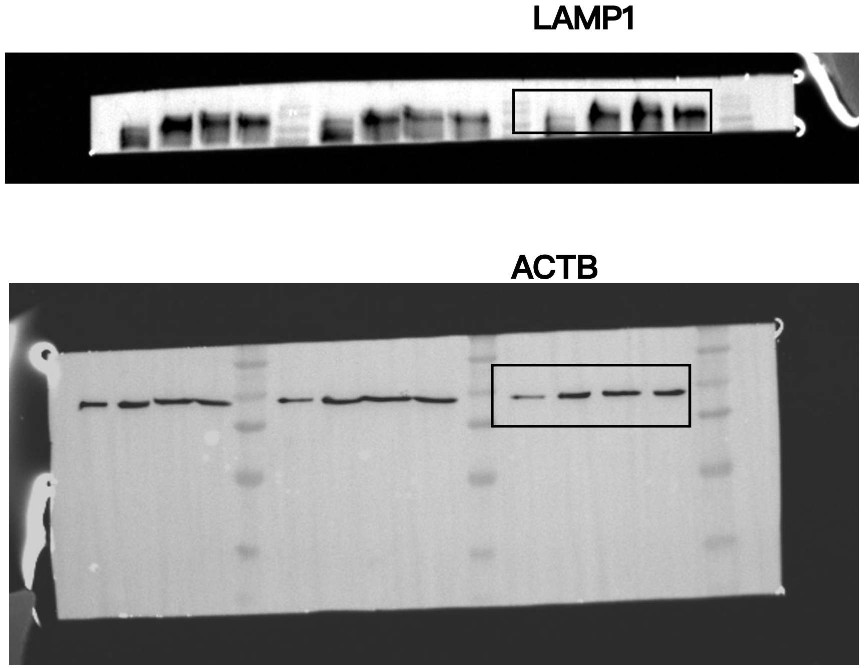

G

Input

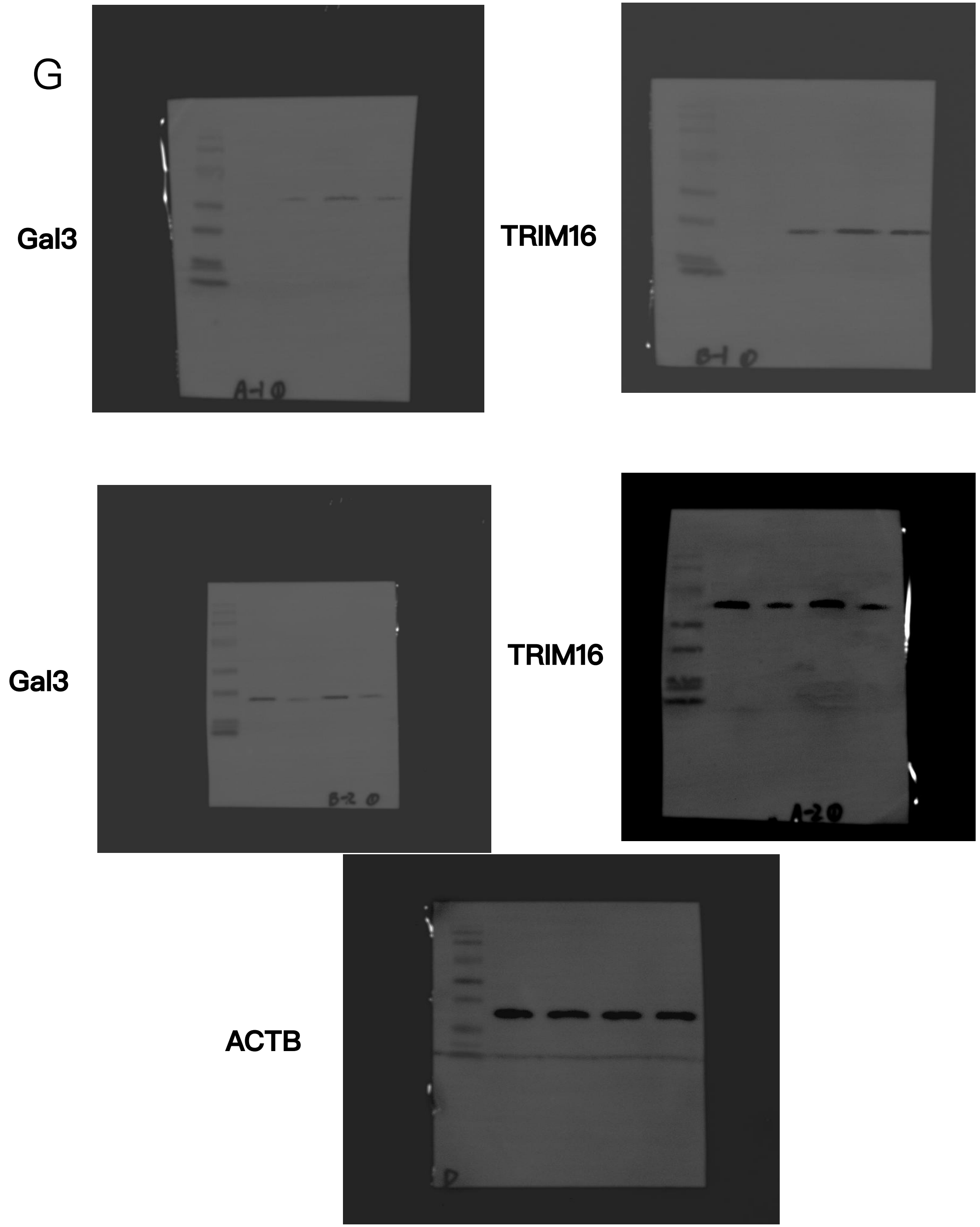

Fig6

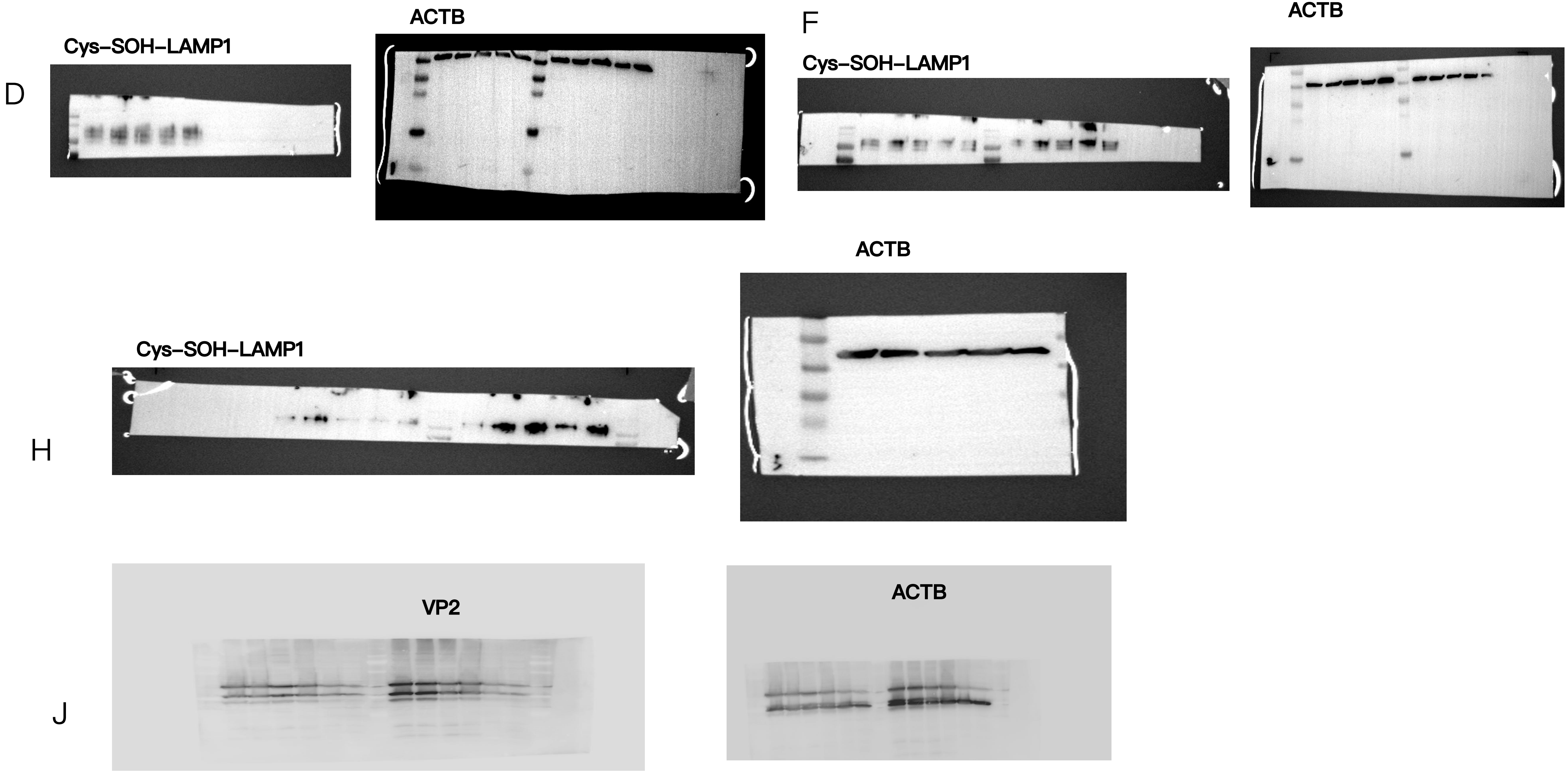
D
Cys-SOH-LAMP1
ACTB
F
Cys-SOH-LAMP1
ACTB
H
Cys-SOH-LAMP1
ACTB
J
VP2
ACTB

Fig7

A

VP2

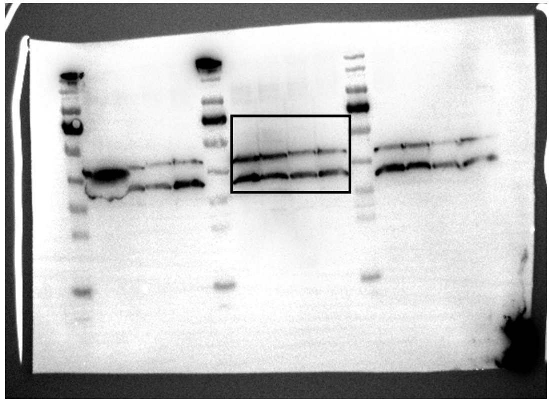

ACTB

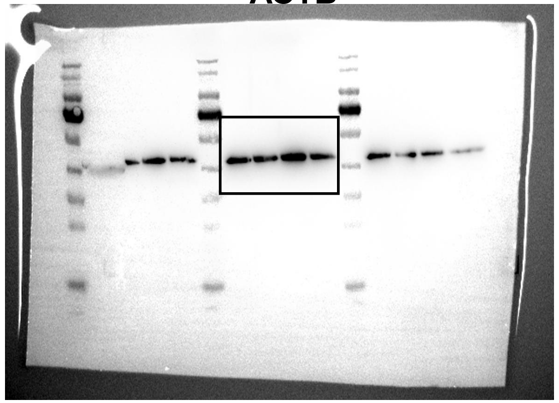

VP2

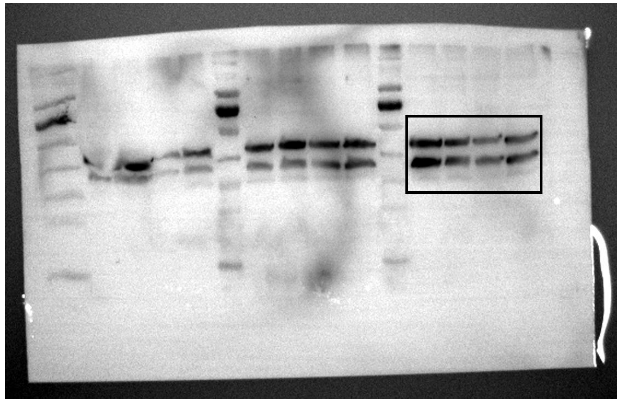

ACTB

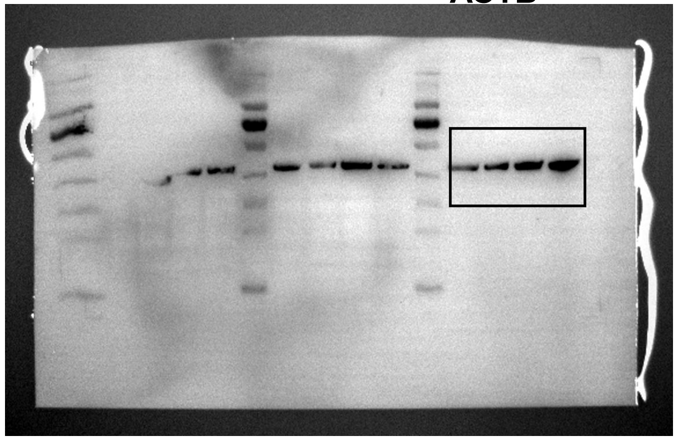

G

LAMP1

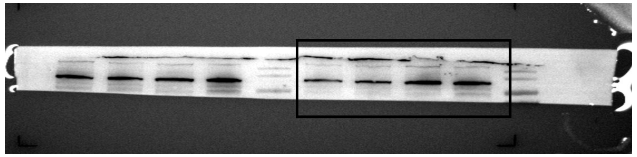

VP2

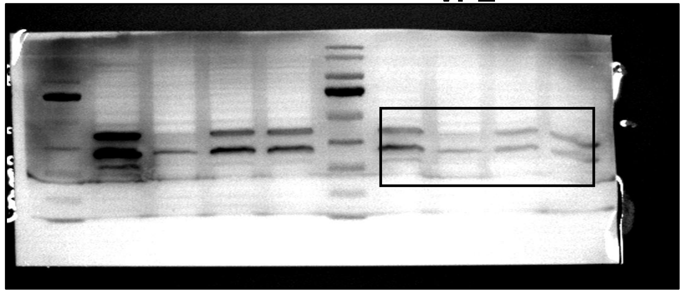

ACTB

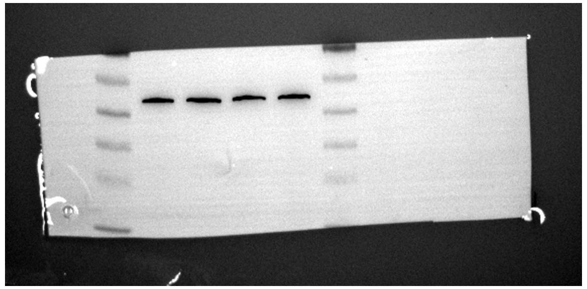

S2

LAMP2

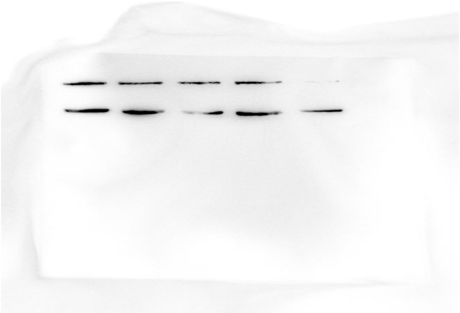

LAMP3

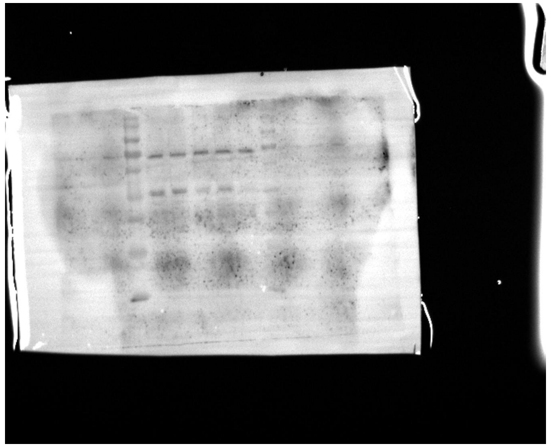

ACTB

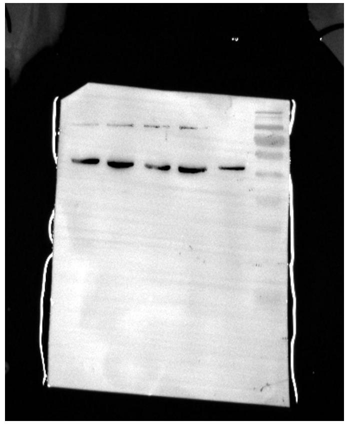

S5

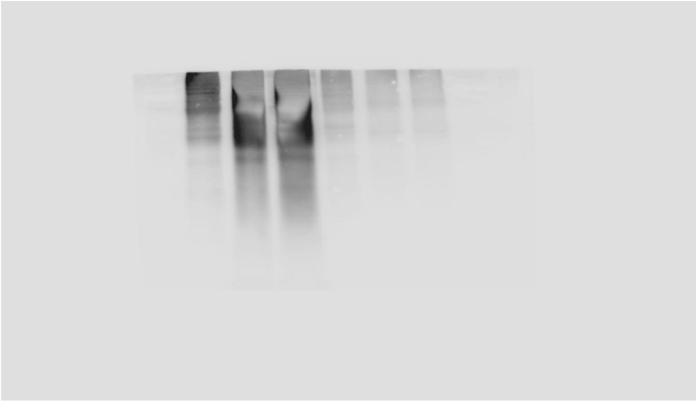

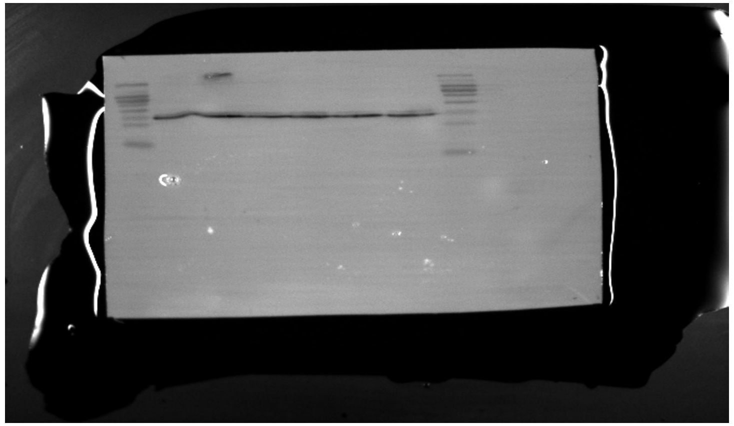

S6

A

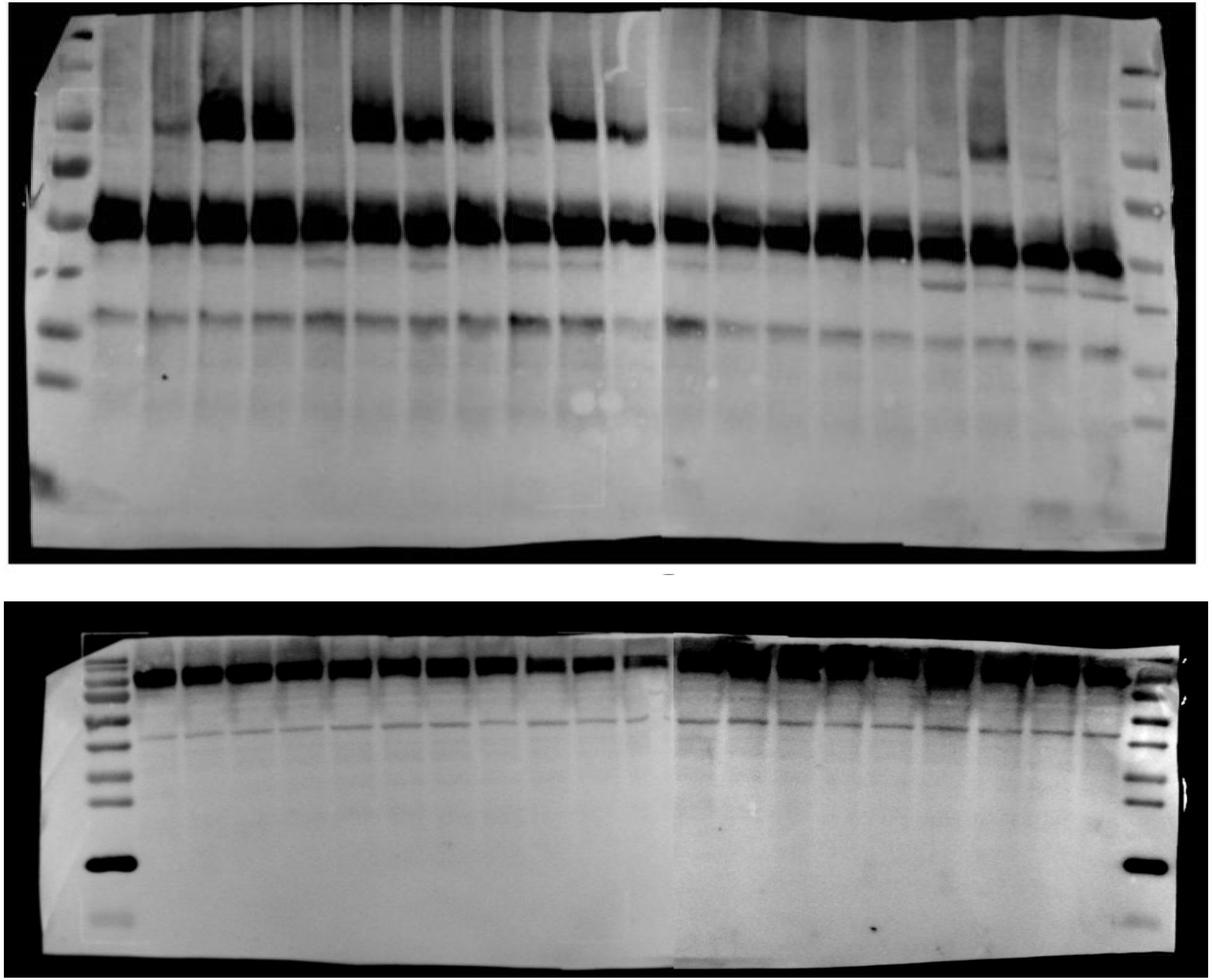

S11

siLAMP1

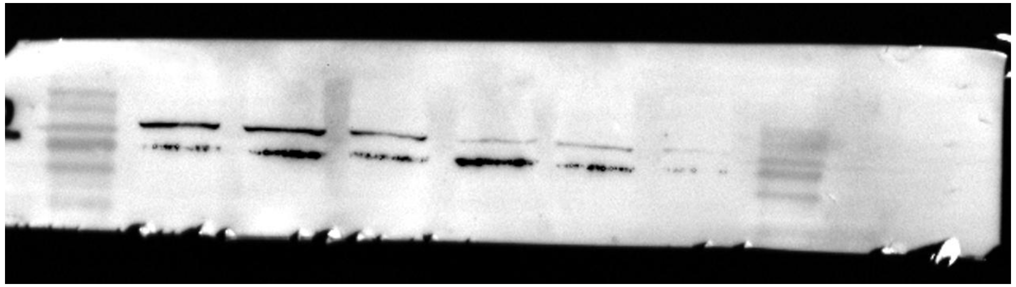

ACTB

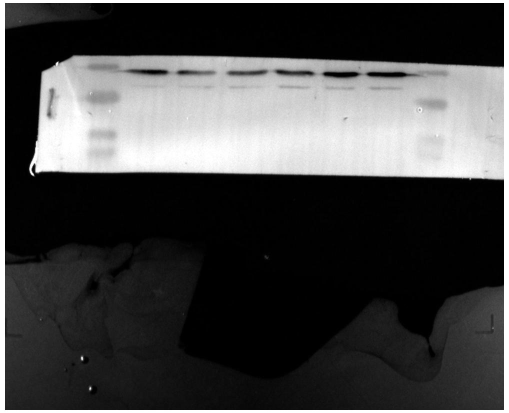

Supplement: S1 Raw Images — (PDF) [file ppat.1013932.s015.pdf]
