# Supplementary material for: A novel SO2 probe inhibits lysophagy induced by Senecavirus A infection by promoting LAMP1 Cys375 sulfenylation
Source: PLoS Pathog. 2026 Feb 5;22(2):e1013932. doi: 10.1371/journal.ppat.1013932 (PMC12875573; doi:10.1371/journal.ppat.1013932)
Supplement: S2 Data — (DOCX) [file ppat.1013932.s017.docx]

Statistical data

Table1.Fig1.B

| 0 h | 3 h | 6 h | 12 h | 24 h |
| --- | --- | --- | --- | --- |
| 0.155018132 | 0.235018132 | 0.488044194 | 0.935672568 | 3.255846308 |
| 0.136347373 | 0.45734777 | 0.558804235 | 0.920774804 | 4.575236262 |
| 0.114346457 | 0.357347124 | 0.5568569222 | 0.945679732 | 3.346547225 |

Table2.Fig1.C

| 0 h | 3 h | 6 h | 12 h | 24 h |
| --- | --- | --- | --- | --- |
| 1.790176301 | 1.476295307 | 1.342979925 | 0.52717778 | 0.46347822 |
| 1.593465763 | 1.543762957 | 1.412467432 | 0.53462768 | 0.42349945 |
| 1.667493224 | 1.456832456 | 1.442979925 | 0.69346645 | 0.44609945 |

Table3.Fig1.D

| 0 h | 3 h | 6 h | 12 h | 24 h |
| --- | --- | --- | --- | --- |
| 0.265826321 | 0.89457998 | 1.194332234 | 1.94574435 | 4.594299034 |
| 0.565826321 | 0.99676979 | 1.383466868 | 2.689319205 | 3.234299024 |
| 0.665826321 | 0.83569799 | 1.294332234 | 1.795693657 | 3.568699434 |

Table4.Fig1.E

| 0 h | 3 h | 6 h | 12 h | 24 h |
| --- | --- | --- | --- | --- |
| 0.443712624 | 0.249789948 | 0.305759963 | 0.244261502 | 0.048714689 |
| 0.734575478 | 0.691653445 | 0.65347114872 | 0.5223423522 | 0.07321145917 |
| 0.824578863 | 0.591634435 | 0.52364557114 | 0.487920835 | 0.0671145917 |

Table5.Fig1.G

|  |  |  |  |  |
| --- | --- | --- | --- | --- |
| 0.0667831231 | 0.031255342 | 0.214671205 | 1.60753932 | 1.842814832 |
| 0.064968848 | 0.051254341 | 0.114686107 | 1.707139932 | 1.433814858 |
| 0.04314968148 | 0.073467561 | 0.1683426146 | 1.5646730964 | 1.723568345 |

Table6.Fig1.H

| 2.431924357 | 2.3938792235 | 2.100345118 | 2.137434706 | 1.84934513421 |
| --- | --- | --- | --- | --- |
| 2.231927458 | 2.171579551 | 2.223412442 | 2.1341345122 | 1.184954487 |
| 2.5678134133 | 2.5023455622 | 2.300846248 | 2.1732342352 | 1.0145954427 |

Table7.Fig1.I

|  |  |  |  |  |
| --- | --- | --- | --- | --- |
| 0.093288881 | 0.138741472 | 0.390102465 | 0.312091133 | 0.584489832 |
| 0.0843283422 | 0.1574567321 | 0.3245645521 | 0.303453223 | 0.43463224 |
| 0.0932567327 | 0.1045723534 | 0.3756134553 | 0.375633223 | 0.3464576333 |

Table8.Fig1.J

|  |  |  |  |  |
| --- | --- | --- | --- | --- |
| 0.047024448 | 0.019697286 | 0.107923383 | 0.047051924 | 0.146745622 |
| 0.037547632 | 0.0749695286 | 0.1062217923 | 0.00345224 | 0.102943213 |
| 0.047024448 | 0.014567242 | 0.107432234 | 0.03464583 | 0.105672223 |

Table9.Fig1.N

| Ctr | SVA |
| --- | --- |
| 0.541 | 0.953 |
| 0.372 | 0.926 |
| 0.631 | 0.877 |
| 0.462 | 0.861 |
| 0.488 | 0.913 |

Table10.Fig2.B

| Ctr | SVA | SVA+DLC | DLC |
| --- | --- | --- | --- |
| 1.035236235 | 0.784631235 | 1.237955878 | 1.315991973 |
| 1.239524632 | 0.823339658 | 1.185101158 | 1.24705638 |
| 1.023523523 | 0.8659578714 | 1.143153414 | 1.267515177 |

Table11.Fig2.D

| 0 | 3 | 6 | 9 | 12 |
| --- | --- | --- | --- | --- |
| 1.403415302 | 1.451356846 | 0.818486633 | 0.524705313 | 0.129098477 |
| 1.603908446 | 1.576899676 | 0.87686633 | 0.470247313 | 0.126897565 |
| 1.776877732 | 1.365899808 | 0.989674654 | 0.524599676 | 0.109679975 |

Table12.Fig2.F

|  |  |  |  |
| --- | --- | --- | --- |
| 2.433443856 | 3.103701833 | 1.086861698 | 1.940280779 |
| 2.180478906 | 3.589097097 | 1.147890867 | 1.40589357 |
| 2.379906653 | 3.898687564 | 1.698979868 | 1.917908763 |

Table13.Fig2.G

| SVA | | SVA+5 μM DLC | |
| --- | --- | --- | --- |
| 1.0032307799 | 1.0582521565 | 1.038015732 | 0.958015732 |
| 0.480382235 | 0.431369523 | 0.465529395 | 0.92299473 |
| 0.385574344 | 0.380443473 | 0.289786585 | 0.538330276 |
| 0.381558839 | 0.447468629 | 0.510029408 | 0.772514876 |
| 0.164270437 | 0.121537317 | 0.194996867 | 0.147942622 |

Table14.Fig2.I

| Ctr | SVA | SVA+DLC | DLC |
| --- | --- | --- | --- |
| 1 | 0.808 | 1.465 | 1.765 |
| 1 | 0.859 | 1.408 | 1.408 |
| 1 | 0.794 | 1.268 | 1.668 |

Table15.Fig2.k

| Ctr | SVA | SVA+DLC | DLC |
| --- | --- | --- | --- |
| 6.902 | 1.075 | 6.874 | 10.661 |
| 6.876 | 1.245 | 6.335 | 9.424 |
| 6.652 | 1.322 | 6.453 | 9.768 |

Table16.Fig2.M

| 1.450164279 | 1.679546985 | 1.195401427 | 0.26721702 | 0.166395059 |
| --- | --- | --- | --- | --- |
| 1.421164279 | 1.679546985 | 1.205401367 | 0.3674302 | 0.266395059 |
| 1.450414279 | 1.6326985 | 1.206101427 | 0.1121621 | 0.260195059 |

Table17.Fig2.N

| 0.358550529 | 0.554795807 | 0.278349634 | 0.072640341 | 5.815894774 |
| --- | --- | --- | --- | --- |
| 0.30155081 | 0.561795917 | 0.488349634 | 0.05264061 | 5.3694725 |
| 0.391550521 | 0.583495807 | 0.27319634 | 0.082640641 | 4.1094774 |

Table18.Fig2.O

| 0.014724661 | 0.082550442 | 0.024492595 | 0.004003756 | 0.011906092 |
| --- | --- | --- | --- | --- |
| 0.034245643 | 0.082548 | 0.023212572 | 0.006782011 | 0.0127606054 |
| 0.024534629 | 0.097646245 | 0.026492217 | 0.0052187037 | 0.0117384142 |

Table19.Fig3.B

| DMSO | Baf A1 | Baf A1+DLC | Rapa | Rapa+DLC | DLC |
| --- | --- | --- | --- | --- | --- |
| 0.846981 | 0.329544 | 0.616519 | 2.49841 | 1.016549 | 0.659841 |
| 0.988112 | 0.59846 | 0.624891 | 2.215849 | 1.06498 | 0.5951 |
| 0.816512 | 0.6269819 | 0.6989749 | 2.3951818 | 1.298455 | 0.68492 |

Table20.Fig3.C

| DMSO | Baf A1 | Baf A1+DLC | Rapa | Rapa+DLC | DLC |
| --- | --- | --- | --- | --- | --- |
| 1.59564 | 4.95819 | 3.98198 | 3.5981 | 2.981981 | 0.8135 |
| 1.216849 | 4.59651 | 3.6591 | 3.9815 | 2.69841 | 0.7884594 |
| 1.3948984 | 4.66849 | 3.65494 | 3.3215 | 2.629815 | 0.8594598 |

Table21.Fig3.E

| DMSO | Baf A1 | Baf A1+DLC | Rapa+DLC | DLC |
| --- | --- | --- | --- | --- |
| 1.38468 | 3.58419 | 1.93546 | 1.222464 | 0.649684 |
| 1.38945 | 3.59455 | 1.78952 | 1.1961635 | 0.69158 |
| 1.356984 | 3.79865 | 1.629848 | 1.1646558 | 0.6981981 |

Table22.Fig3.G

| Ctr | SVA | SVA+2 DLC | SVA+5DLC |
| --- | --- | --- | --- |
| 3.096187916 | 0.805504506 | 0.863714397 | 0.547387219 |
| 2.035621496 | 0.83056506 | 0.626637297 | 0.593215652 |
| 3.120325593 | 0.57640515 | 0.81335697 | 0.522695949 |

Table23.Fig3.H

| Ctr | SVA | SVA+2 DLC | SVA+5DLC |
| --- | --- | --- | --- |
| 0.019167581 | 1.082516925 | 1.346550093 | 0.878189538 |
| 0.011959251 | 1.0369416925 | 1.2694550693 | 0.9578189538 |
| 0.015129541 | 1.3682516925 | 1.358548593 | 0.8826418738 |

Table24.Fig3.i

| Ctr | SVA | SVA+2DLC | SVA+5DLC |
| --- | --- | --- | --- |
| 1.02 | 3.51 | 3.03 | 3.15 |
| 1.01 | 3.53 | 3.12 | 3.21 |
| 1.12 | 3.55 | 3.15 | 3.35 |

Table25.Fig4.C

| 0.23 | 0.87 | 0.72 | 2.01 | 0.76 | 1.55 |
| --- | --- | --- | --- | --- | --- |
| 0.25 | 0.88 | 0.73 | 1.9 | 0.63 | 1.54 |
| 0.3 | 0.91 | 0.61 | 1.87 | 0.66 | 1.81 |

Table26.Fig4.E

| SVA | DLC | MG132 | DLC+MG132 |
| --- | --- | --- | --- |
| 0.2413574 | 0.4474422 | 0.510385786 | 0.538039 |
| 0.2313439 | 0.5971702 | 0.614524964 | 0.539690 |
| 0.2534663 | 0.5146714 | 0.631345611 | 0.634651 |

Table27.Fig4.H

| Ctr | SVA+DLC | SVA |
| --- | --- | --- |
| 0.67 | 0.781 | 0.94 |
| 0.69 | 0.75585 | 0.95 |
| 0.68 | 0.8727 | 0.92 |

Table28.Fig5.B

| 0 hpi | 24 hpi |
| --- | --- |
| 1.951844262 | 0.912866613 |
| 2.48042435 | 0.855912679 |
| 2.455177276 | 0.931927789 |
|  |  |
|  |  |

Table29.Fig5.D

| 0.935 | 0.625 | 0.933 |
| --- | --- | --- |
| 0.915 | 0.594 | 0.945 |
| 0.907 | 0.644 | 0.955 |

Table30.Fig5.F

|  |  |  |
| --- | --- | --- |
| 13 | 3 | 13 |
| 15 | 2 | 14 |
| 18 | 4 | 15 |
| 14 | 5 | 14 |
| 15 | 4 | 15 |
| 17 | 4 | 13 |
| 14 | 3 | 15 |
| 15 | 3 | 16 |
| 18 | 4 | 14 |
| 15 | 4 | 13 |

Table31.Fig6.E

| Ctr | DLC | SVA | SVA+DLC | SVA+DLC+DTT |
| --- | --- | --- | --- | --- |
| 0.465327668 | 0.398543331 | 0.259865834 | 0.292266905 | 0.06515198 |
| 0.455044256 | 0.386278017 | 0.20455429 | 0.390253566 | 0.283140782 |
| 0.404921899 | 0.549163184 | 0.24213662 | 0.324622806 | 0.214684 |

Table32.Fig6.G

| SVA+DMSO | SVA+DLC+SO_2_ | SVA+DLC | SVA+DLC+SO_2_+DTT |
| --- | --- | --- | --- |
| 0.307594446 | 1.351007094 | 0.68485126 | 1.061932663 |
| 0.517916255 | 1.267235634 | 0.563429837 | 0.560275649 |
| 0.177131668 | 1.355377777 | 0.5320651961 | 1.091940779 |

Table33.Fig6.I

| SVA | SVA+DLC | C375ASVA | C375ASVA+DLC | C375ASVA+DLC+DTT |
| --- | --- | --- | --- | --- |
| 0.328442199 | 0.299598294 | 0.61594876 | 0.893881892 | 0.25540697 |
| 0.045484383 | 0.031009147 | 0.429428912 | 0.771317693 | 0.094243169 |
| 0.315442199 | 0.199598294 | 0.51594876 | 0.793881892 | 0.15540697 |

Table34.Fig6.K

| 0 | 50 | 100 | 250 | 500 | 1000 |
| --- | --- | --- | --- | --- | --- |
| 2.52755012 | 2.02930349 | 1.58677634 | 0.92948045 | 0.39224568 | 0.20223456 |
| 2.53546452 | 2.24572457 | 1.33563562 | 0.93568833 | 0.33784699 | 0.25689677 |
| 2.34613463 | 2.11234123 | 1.12354513 | 0.82122131 | 0.42342944 | 0.23521344 |

Table35.Fig7.B

| 0 | 2 | 5 | 10 |
| --- | --- | --- | --- |
| 1.6427282 | 1.263871837 | 0.976068358 | 0.654625894 |
| 1.834044296 | 1.239892996 | 1.015719607 | 0.64287283 |
| 1.460176495 | 1.317472153 | 1.049587376 | 0.525881847 |

Table36.Fig7.C

| 0 | 2 | 5 | 10 |
| --- | --- | --- | --- |
| 1.276872332 | 1.141322091 | 0.977465125 | 0.995734974 |
| 1.4045074839 | 1.02439424 | 0.920528819 | 0.65758915 |
| 1.185937102 | 1.125033615 | 0.904231657 | 0.707594164 |

Table37.Fig7.D

| SVA | DLC1μM+SVA | DLC 2μM+SVA | DLC 5μM+SVA |
| --- | --- | --- | --- |
| 8.54 | 8.21 | 7.54 | 7.21 |
| 9.24 | 8.33 | 7.65 | 7.35 |
| 8.67 | 8.05 | 7.94 | 7.52 |

Table38.Fig7.E

| SVA | DLC1μM+SVA | DLC 2μM+SVA | DLC 5μM+SVA |
| --- | --- | --- | --- |
| 8.54 | 8.21 | 8.54 | 7.21 |
| 9.24 | 9.33 | 7.65 | 8.35 |
| 8.67 | 9.05 | 7.94 | 7.52 |

Table39.Fig7.H,I

| 2.24 | 0.345 | 0.253 | 0.238 |
| --- | --- | --- | --- |
| 2.53 | 0.392 | 0.238 | 0.256 |
| 2.245 | 0.441 | 0.221 | 0.251 |

|  |  |  |  |
| --- | --- | --- | --- |
| 0.784066303 | 1.40046803 | 1.90566196 | 1.723905091 |
| 0.629841 | 1.529723266 | 1.9525418541 | 1.928418952 |
| 0.7182268 | 1.458123722 | 1.8549526165 | 1.828741816 |

Table40.Fig7.K,L

| SVA | SVA+DLC |
| --- | --- |
| 0.91 | 0.65 |
| 0.88 | 0.54 |
| 0.87 | 0.55 |

| SVA | SVA+DLC |
| --- | --- |
| 0.35 | 0.44 |
| 0.45 | 0.45 |
| 0.47 | 0.35 |

Table41.Fig8.B

| SVA(1×10^-7^TCID_50_) | SVA(1×10^-7^TCID_50_)+2mg/kg DLC | SVA(1×10^-7^TCID_50_)+5mg/kg DLC |
| --- | --- | --- |
| 4.469822016 | 4.338456494 | 3.712649702 |
| 4.478566496 | 4.100370545 | 3.761175813 |
| 4.477121255 | 4.274157849 | 3.698100546 |

| SVA(1×10^-7^TCID_50_) | SVA(1×10^-7^TCID_50_)+2mg/kg DLC | SVA(1×10^-7^TCID_50_)+5mg/kg DLC |
| --- | --- | --- |
| 4.477121255 | 3.906335042 | 4.457881897 |
| 4.482873584 | 3.8876173 | 3.894869657 |
| 4.469822016 | 3.82672252 | 3.73479983 |

| SVA(1×10^-7^TCID_50_) | SVA(1×10^-7^TCID_50_)+2mg/kg DLC | SVA(1×10^-7^TCID_50_)+5mg/kg DLC |
| --- | --- | --- |
| 4.481442629 | 4.336459734 | 4.025305865 |
| 4.494154594 | 4.089905111 | 4.278753601 |
| 4.57054294 | 4.414973348 | 4.346352974 |

| SVA(1×10^-7^TCID_50_) | SVA(1×10^-7^TCID_50_)+2mg/kg DLC | SVA(1×10^-7^TCID_50_)+5mg/kg DLC |
| --- | --- | --- |
| 4.488550717 | 4.380211242 | 4.201397124 |
| 4.474216264 | 3.880813592 | 3.859138297 |
| 4.474216264 | 4.245512668 | 3.73239376 |

| SVA(1×10^-7^TCID_50_) | SVA(1×10^-7^TCID_50_)+2mg/kg DLC | SVA(1×10^-7^TCID_50_)+5mg/kg DLC |
| --- | --- | --- |
| 4.459392488 | 3.744292983 | 3.767897616 |
| 4.456366033 | 3.677606953 | 3.919601024 |
| 4.491361694 | 3.59439255 | 3.701567985 |

| SVA(1×10^-7^TCID_50_) | SVA(1×10^-7^TCID_50_)+2mg/kg DLC | SVA(1×10^-7^TCID_50_)+5mg/kg DLC |
| --- | --- | --- |
| 4.469822016 | 4.478566496 | 3.694605199 |
| 4.602059991 | 3.568201724 | 3.684845362 |
| 4.519827994 | 4.170261715 | 3.670245853 |

Table42.Fig8.C

| PBS | SVA（1×10^-7^TCID_50_) | SVA（2×10^-7^TCID_50_) | SVA（1×10^-7^TCID_50_)+DLC（2 mg/kg) | SVA（2×10^-7^TCID_50_)+DLC（2 mg/kg) |
| --- | --- | --- | --- | --- |
| 0.157142857 | 0.02 | 0.035 | 0.055 | 0.045 |
| 0.147619048 | 0.035 | 0 | 0.072 | 0.035 |
| 0.10952381 | 0.025 | 0.015 | 0.076 | 0.055 |
| 0.171428571 | 0.025 | 0.01 | 0.075 | 0.08 |
| 0.123809524 | 0.05 | 0.02 | 0.055 | 0.039 |

Table43.FigS2

LAMP2

| 0 | 3 | 6 | 12 | 24 |
| --- | --- | --- | --- | --- |
| 0.171901365 | 0.174144868 | 0.233838151 | 0.220111159 | 0.7095627 |
| 0.123994732 | 0.12275978 | 0.321147549 | 0.537988539 | 0.429332053 |
| 0.127553766 | 0.120355883 | 0.311122994 | 0.679060387 | 1.018302971 |

LAMP3

| 0 | 3 | 6 | 12 | 24 |
| --- | --- | --- | --- | --- |
| 0.75123 | 0.5342 | 0.5324 | 0.4526 | 0.24562 |
| 0.58313 | 0.5721 | 0.5624 | 0.4231 | 0.2834 |
| 0.6215 | 0.5441 | 0.5382 | 0.4622 | 0.2442 |

Table44.FigS3

| 0 | 3 | 6 | 12 | 24 |
| --- | --- | --- | --- | --- |
| 145.7802321 | 110.2913713 | 105.3305907 | 85.9661498 | 93.5752532 |
| 138.2455134 | 110.23487233 | 101.12872341 | 90.3234215 | 81.13452131 |
| 128.5752532 | 123.9661498 | 103.3305907 | 93.1234321 | 85.28698313 |

| 0 | 3 | 6 | 12 | 24 |
| --- | --- | --- | --- | --- |
| 91.46177217 | 100.2043566 | 105.2175949 | 119.6919409 | 130.3242722 |
| 85.28698313 | 100.3105485 | 117.0405485 | 123.2153375 | 135.1779008 |
| 85.18739313 | 105.3242722 | 111.2175949 | 117.4154274 | 148.5752532 |
|  |  |  |  |  |

Table45.FigS4

| 0 | 0.5 | 1 | 2.5 | 5 | 10 |
| --- | --- | --- | --- | --- | --- |
| 1 | 0.917582725 | 0.895979115 | 0.879071845 | 0.891875796 | 0.841791089 |
| 1 | 0.941340392 | 0.961063309 | 0.834064253 | 0.899808577 | 0.896165708 |
| 1 | 0.995979115 | 0.941340392 | 0.921875796 | 0.894064253 | 0.899808577 |

| 0 | 0.5 | 1 | 2.5 | 5 | 10 |
| --- | --- | --- | --- | --- | --- |
| 1 | 0.981440192 | 0.900864373 | 0.940169916 | 0.910727554 | 0.738705037 |
| 1 | 0.943172801 | 0.903036101 | 0.900033538 | 0.847632218 | 0.805568786 |
| 1 | 0.878974675 | 0.842821885 | 0.902915635 | 0.800687363 | 0.867037483 |

Table46.FigS5

| BTD-MOCK | BTD-SVA | BTD-SVA-DLC | DMSO-MOCK | DMSO-SVA | DMSO-SVA-DLC |
| --- | --- | --- | --- | --- | --- |
| 14.80255148 | 9.105663463 | 17.89696977 | 1.126010697 | 0.958882921 | 0.936674248 |
| 10.204130115 | 9.891819611 | 17.62600985 | 2.079367697 | 1.332148468 | 1.083774442 |
| 11.34516074 | 5.859107961 | 13.73521552 | 1.270701931 | 1.101736248 | 1.210129726 |

Table47.FigS6

|  |  |
| --- | --- |
| 1 | 0.7 |
| 1 | 0.8 |
| 1 | 0.65 |

| 1 | 0.8 |
| --- | --- |
| 1 | 0.7 |
| 1 | 0.9 |

|  |  |
| --- | --- |
| 1 | 0.3 |
| 1 | 0.4 |
| 1 | 0.4 |

Table48.FigS10

| SVA | 2μM | 5μM | 10μM |
| --- | --- | --- | --- |
| 0.855699992 | 0.598300004 | 0.50170002 | 0.52249999 |
| 0.803000009 | 0.538900006 | 0.405100024 | 0.569899988 |
| 0.722500026 | 0.490700012 | 0.494199991 | 0.488700008 |

Table49.FigS11

| Scramble10 | 30 | 50 | siRNA 10 | 30 | 50 |
| --- | --- | --- | --- | --- | --- |
| 1.06609249 | 1.081147919 | 1.053175065 | 0.49096649 | 0.0829644081 | 0.035998267 |
| 1.205291533 | 1.09422263 | 1.0823976207 | 0.614098093 | 0.121824758 | 0.133413982 |
| 1.238989705 | 1.0779894286 | 1.0558776432 | 0.490471341 | 0.043827893 | 0.050614062 |

Table50.FigS12

|  |  | AREA |  |  |  |  |  | %AREA |  |  |  |  |
| --- | --- | --- | --- | --- | --- | --- | --- | --- | --- | --- | --- | --- |
| 1 | 肝sva-1.jpg:Red | 449624 | 0.119 | 0.643 | 467.419 | 351.726 | 3498 | 84604.761 | 75096461 | 0.643 | 0.119 | 0.188167805 |
| 1 | 肝sva-2.jpg:Red | 365922 | 0.159 | 1.327 | 486.049 | 371.354 | 3498 | 80235.19 | 56892750 | 1.327 | 0.159 | 0.21926856 |
| 1 | 肝sva-3.jpg:Red | 398214 | 0.112 | 0.794 | 675.21 | 396.9 | 3498 | 80855.991 | 64398849 | 0.794 | 0.112 | 0.20304658 |
| 1 | 肝高-3.jpg:Red | 371868 | 0.128 | 0.225 | 515.713 | 357.987 | 3500 | 64568.943 | 63698675 | 0.225 | 0.128 | 0.173634039 |
| 1 | 肝高-2.jpg:Red | 364814 | 0.128 | 0.251 | 574.88 | 399.771 | 3500 | 66625.953 | 61271170 | 0.251 | 0.128 | 0.182629924 |
| 1 | 肝高-1.jpg:Red | 486961 | 0.186 | 0.691 | 485.823 | 382.908 | 3500 | 94860.707 | 50108954 | 0.691 | 0.186 | 0.194801446 |
|  |  |  |  |  |  |  |  |  |  |  |  |  |
| 1 | 肺sva-1.jpg:Red | 150559 | 0.114 | 0.407 | 473.621 | 409.908 | 3498 | 31112.808 | 24195103 | 0.407 | 0.114 | 0.20664861 |
| 1 | 肺sva-2.jpg:Red | 119248 | 0.114 | 0.447 | 596.478 | 408.345 | 3498 | 24842.346 | 19142905 | 0.447 | 0.114 | 0.208325054 |
| 1 | 肺sva-3.jpg:Red | 154016 | 0.121 | 0.827 | 533.484 | 350.775 | 3498 | 35745.543 | 23655878 | 0.827 | 0.121 | 0.232089802 |
| 1 | 肺高-1.jpg:Red | 131354 | 0.146 | 0.317 | 486.879 | 380.499 | 3498 | 28798.349 | 20345899 | 0.317 | 0.146 | 0.219242269 |
| 1 | 肺高-2.jpg:Red | 114393 | 0.119 | 0.23 | 355.154 | 417.544 | 3498 | 19239.842 | 19859891 | 0.23 | 0.119 | 0.168190728 |
| 1 | 肺高-3.jpg:Red | 214246 | 0.142 | 0.526 | 488.782 | 382.991 | 3498 | 34702.894 | 30971713 | 0.526 | 0.142 | 0.161976858 |
|  |  |  |  |  |  |  |  |  |  |  |  |  |
| 1 | 心sva-1.jpg:Red | 433079 | 0.095 | 0.543 | 562.436 | 346.744 | 3498 | 60978.174 | 80349864 | 0.543 | 0.095 | 0.140801503 |
| 1 | 心sva-2.jpg:Red | 369097 | 0.076 | 0.248 | 656.108 | 379.855 | 3498 | 46072.177 | 70827390 | 0.248 | 0.076 | 0.124824035 |
| 1 | 心sva-3.jpg:Red | 423085 | 0.101 | 0.411 | 599.252 | 355.902 | 3498 | 66819.281 | 75491191 | 0.411 | 0.101 | 0.157933467 |
| 1 | 高心-3.jpg:Red | 165736 | 0.139 | 0.794 | 410.331 | 359.935 | 3498 | 32744.482 | 27110447 | 0.794 | 0.139 | 0.197570124 |
| 1 | 高心-2.jpg:Red | 297175 | 0.119 | 0.394 | 409.918 | 412.328 | 3498 | 50001.858 | 51833352 | 0.394 | 0.119 | 0.168257283 |
| 1 | 高心-1.jpg:Red | 139900 | 0.156 | 0.929 | 424.456 | 365.999 | 3498 | 33506.439 | 20971362 | 0.929 | 0.156 | 0.239502781 |
|  |  |  |  |  |  |  |  |  |  |  |  |  |
| 1 | 脾sva-1.jpg:Red | 260829 | 0.128 | 0.276 | 599.498 | 403.554 | 3498 | 54717.352 | 41225554 | 0.276 | 0.128 | 0.209782471 |
| 1 | 脾sva-2.jpg:Red | 269470 | 0.119 | 0.26 | 534.337 | 414.547 | 3498 | 52374.441 | 44117261 | 0.26 | 0.119 | 0.194360934 |
| 1 | 脾sva-3.jpg:Red | 311560 | 0.119 | 0.276 | 439.984 | 383.306 | 3498 | 63582.524 | 49928972 | 0.276 | 0.119 | 0.204077943 |
| 1 | 脾高-1.jpg:Red | 273691 | 0.213 | 0.289 | 515.049 | 382.226 | 3500 | 44191.345 | 24687655 | 0.289 | 0.213 | 0.16146437 |
| 1 | 脾高-2.jpg:Red | 151876 | 0.135 | 0.248 | 541.883 | 390.627 | 3500 | 30157.684 | 24587947 | 0.248 | 0.135 | 0.198567805 |
| 1 | 脾高-3.jpg:Red | 94217 | 0.126 | 0.222 | 579.481 | 317.947 | 3500 | 16837.395 | 15954612 | 0.222 | 0.126 | 0.178708673 |
|  |  |  |  |  |  |  |  |  |  |  |  |  |
| 1 | 肾sva-1.jpg | 407167 | 0.171 | 0.498 | 501.402 | 375.244 | 3500 | 93660.878 | 61613480 | 0.498 | 0.171 | 0.230030621 |
| 1 | 肾sva-2.jpg | 372709 | 0.121 | 0.26 | 612.35 | 383.776 | 3500 | 68248.717 | 62559136 | 0.26 | 0.121 | 0.183115291 |
| 1 | 肾sva-3.jpg | 489325 | 0.128 | 0.682 | 580.704 | 393.949 | 3500 | 110036.139 | 75375490 | 0.682 | 0.128 | 0.224873323 |
| 1 | 肾高-1.jpg | 419560 | 0.205 | 0.992 | 457.19 | 389.218 | 3498 | 83396.029 | 44993676 | 1.009 | 0.205 | 0.198770209 |
| 1 | 肾高-2.jpg | 303962 | 0.114 | 0.2 | 522.046 | 396.887 | 3498 | 49514.429 | 53347858 | 0.2 | 0.114 | 0.162896773 |
| 1 | 肾高-3.jpg | 376648 | 0.208 | 1.293 | 560.545 | 380.01 | 3498 | 74027.064 | 38437584 | 1.327 | 0.208 | 0.196541768 |
